# Transcriptomic profile of the predatory mite *Amblyseius swirskii* (Acari: Phytoseiidae) on different host plants

**DOI:** 10.1007/s10493-022-00715-w

**Published:** 2022-05-09

**Authors:** Angeliki Paspati, Alberto Urbaneja, Joel González-Cabrera

**Affiliations:** 1 Centro de Protección Vegetal y Biotecnología, Unidad Mixta Gestión Biotecnológica de Plagas UV-IVIA, Instituto Valenciano de Investigaciones Agrarias (IVIA), Carretera Moncada-Náquera km 4,5, Moncada, 46113 Valencia, Spain; 2 Department of Genetics, Institute BIOTECMED, Unidad Mixta Gestión Biotecnológica de Plagas UV-IVIA, Universitat de València, Dr Moliner 50, Burjassot, 46100 Valencia, Spain; 3 Present Address: HAO-DEMETER, Institute of Olive, Subtropical Crops and Viticulture, IOSV, Heraklion, Greece

**Keywords:** Acyl sugar, Detoxification, Differential expression, Phytoseiid, RNAseq, Trichome, Tomato, Exudate, Pepper

## Abstract

*Amblyseius swirskii* Athias-Henriot (Acari: Phytoseiidae) is a predatory mite, effective at controlling whiteflies and thrips in protected crops. However, on tomato its efficacy as a biocontrol agent is hindered, most probably by the plant trichomes and their exudates. Our aim was to characterize the response of *A. swirskii* to the tomato trichome exudates and identify three major detoxification gene sets in this species: cytochromes P450 (CYPs), glutathione *S*-transferases (GSTs) and carboxyl/cholinesterases (CCEs). Mites were exposed separately to tomato and pepper, a favourable host plant for *A. swirskii*, after which their transcriptional responses were analysed and compared. The de novo transcriptome assembly resulted in 71,336 unigenes with 66.1% of them annotated. Thirty-nine *A. swirskii* genes were differentially expressed after transfer on tomato leaves when compared to pepper leaves; some of the expressed genes were associated with the metabolism of tomato exudates. Our results illustrate that the detoxification gene sets CYPs, GSTs and CCEs are abundant in *A. swirskii*, but do not play a significant role when in contact with the tomato exudates.

**Supplementary Information:**

The online version contains supplementary material available at 10.1007/s10493-022-00715-w.

## Introduction

Tomato (*Solanum lycopersicum*) is one of the most important vegetables cultivated worldwide, with over 180 million tonnes produced in 2019 (FAOSTAT 2021), but high yield losses are reported annually, due to damage from various pests, including the specialists of Solanaceae: the red tomato spider mite *Tetranychus evansi* (Baker & Pritchard) (Acari: Tetranychidae), the tomato russet mite *Aculops lycopersici* (Massee) (Acari: Eriophyidae) and the South American tomato pinworm *Tuta absoluta* (Meyrick) (Lepidoptera: Gelechiidae) (Pérez-Hedo et al. 2017). In addition to the specialists, other generalist herbivores such as the two spotted spider mite *Tetranychus urticae* Koch (Acari: Tetranychidae) and the cotton whitefly *Bemisia tabaci* (Gennadius) (Hemiptera: Aleyrodidae) are listed as major tomato pests (Pérez-Hedo et al. 2017).

Integrated pest management (IPM) is currently the most effective strategy to fight pests in many protected horticultural crops (van Lenteren et al. 2018). It relies vastly on biological control approaches, including the use of predatory mites. However, on tomato, predatory mites are not effective due to the difficulty in establishing on this crop (e.g., Castagnoli et al. 1999; Cédola and Sánchez 2003).

In tomato, the survival and efficacy of small predators is hindered by plant defences mediated by the trichomes and their exudates (van Haren et al. 1987; Nihoul 1994; Cédola et al. 2001; Cédola and Sánchez 2003; Paspati et al. 2021). Tomato trichomes are classified in seven categories (types I–VII) according to their morphological and physiological properties. Types I, IV, VI and VII are glandular and II, III and V are non-glandular (Luckwill 1943). Non-glandular trichomes are thought to mainly function as a physical barrier to arthropod feeding and movement, obstructing herbivore dispersal throughout the plant surface (Baur et al. 1991; Simmons and Gurr 2005). Glandular trichomes are characterized by the heads present on their tips, producing sticky, toxic, compounds that may cause lethal entrapment, acute toxicity, or repellence of arthropods (Simmons and Gurr 2005; Antonious and Snyder 2006; Glas et al. 2012). Moreover, tomato glandular trichomes and their exudates may have profound effects on herbivore performance (i.e., growth, survival and fecundity) (Duffey and Isman 1981; Cédola and Sánchez 2003; Kennedy 2003; Leckie et al. 2016). A variety of secondary metabolites are secreted by the tomato glandular trichomes, including the sticky acyl sugars which are the most abundant chemical group, followed by terpenoids, phenols, methyl ketones and others (Schilmiller et al. 2010). In cultivated tomato*,* the acyl sugars are produced and excreted by the most abundant glandular trichomes, type I and VI (Kang et al. 2010; Ghosh and Jones 2017; Paspati et al. 2021). The antibiotic and antixenotic effects of these metabolites render resistance against herbivores but also affect negatively natural enemies (Simmons and Gurr 2005).

Phytophagous mites, including the specialists *T. evansi*, *A. lycopersici* and the generalist *T. urticae*, have adapted to tomato defences and in some cases evolved mechanisms that suppress them. For example, *A. lycopersici* triggers the degradation of the glandular trichomes (van Houten et al. 2013), whereas *T. evansi* suppresses the signalling pathways of the phytohormones jasmonic acid and salicylic acid (Alba et al. 2015). *Tetranychus urticae* suppresses the defence downstream of these phytohormones and also modifies the expression of various families of detoxification genes (Alba et al. 2015; Grbić et al. 2011; Dermauw et al. 2013; Wybouw et al. 2015). Interestingly, the genome of *T. urticae* is characterized by striking species-specific expansions of gene families associated with detoxification (Grbić et al. 2011). Furthermore, genes acquired by lateral gene transfer from fungi and bacteria, associated with digestion and detoxification, have been identified in *T. urticae* (Grbić et al. 2011).

*Amblyseius swirskii* Athias-Henriot (Acari: Phytoseiidae) is a polyphagous predatory mite, which is used in augmentative biological control of whiteflies (greenhouse whiteflies, silverleaf whiteflies), thrips (western flower thrips, onion thrips, chilli thrips), and plant-feeding mites (spider mites, broad mite) in many vegetable, fruit, and flower crops (e.g., Stansly and Castillo 2010; Calvo et al. 2011; Hoogerbrugge et al. 2011). On detached tomato leaflets, *A. swirskii* can attack and reproduce using common tomato pests, such as *A. lycopersici* (Momen and Abdel-Khalek 2008), *T. absoluta* (Momen et al. 2013), *B. tabaci* and *T. urticae* (personal observation). However, the density of glandular trichomes is much higher on tomato stems where the predatory mites often get trapped and die (van Haren et al. 1987; Paspati et al. 2021).

Our aim is to characterize the transcriptomic response of *A. swirskii* to the tomato trichome exudates and identify genes belonging to three protein superfamilies known to be involved in detoxification processes: the cytochromes P450 monooxygenases (CYPs), the glutathione S-transferases (GSTs) and the carboxyl/cholinesterases (CCEs). The number of these genes present in arthropods has been correlated with their adaptation to toxic plant allelochemicals (Heidel-Fischer and Vogel 2015).

## Experimental procedures

### Plant and mites

Tomato plants (*S. lycopersicum* cv. Raf Marmande) and pepper plants (*Capsicum annuum* cv. Lipari) were used to determine the metabolic responses of *A. swirskii* to both host plants. Seeds were sown in a mixture of soil and local peat moss. Two weeks after germination seedlings were individually transplanted into pots (8 × 8 × 8 cm). Plants were maintained undisturbed at 25 ± 2 ºC, 65 ± 5% relative humidity and L14:D10 h photoperiod. Pesticide-free plants with six fully developed leaves (approximately 20 cm in height) were used for the experiments at 4 weeks of age.

Colonies of *A. swirskii* were initiated from specimens supplied by Koppert Biological Systems (Águilas, Murcia, Spain) and were maintained in rearing units which consisted of a piece of hard black plastic placed on a water-saturated sponge (based on Overmeer 1981). Twice a week, mites were fed ad libitum with *Carpobrotus edulis* pollen (Aizoaceae) (Ragusa and Swirski 1975). The colonies were maintained at 25 ± 2 ºC in growth chambers under a L16:D8 photoperiod and 80% RH for approximately 2 months (approx. four generations).

### Exposure to tomato and pepper, sample collection

Thirty *A. swirskii* females were placed on top of five tomato or pepper leaf discs (5 cm diameter) and left undisturbed for 48 h with no other food source. The leaf discs were placed on top of water-saturated sponges, inside a plastic tray filled with water. The borders of the leaf disc were covered with water-saturated tissue paper to ensure a constant water supply for the phytoseiids, and to prevent them from escaping. Before the release, the tomato leaf discs were prepared from the middle leaf of a plant (third leaf) and were observed under the stereoscope to confirm the presence of glandular trichomes, mostly type VI. As the predatory mites walk on the tomato leaves, the trichome glandular heads break, releasing the exudates. Female mites of different ages were released on the adaxial surface of the leaf disc. Fourteen female mites were collected from each leaf disc with a fine paint brush. Thus, 70 female mites per crop were collected in microcentrifuge tubes. They were snap frozen in liquid nitrogen and stored at −80 ºC until used. This process was replicated 4× (four biological replicates per crop).

### RNA extraction, quantification and qualification

Total RNA from the eight biological replicates was extracted using the RNeasy mini kit (Qiagen, Hilden, Germany) according to the manufacturer recommendations. RNA degradation and contamination were monitored by electrophoresis in 1% agar gels. RNA concentration was measured using Qubit® RNA Assay Kit in Qubit v.2.0 Fluorometer (Life Technologies, Carlsbad, CA, USA). RNA integrity was assessed using the RNA Nano 6000 Assay Kit of the Agilent Bioanalyzer 2100 system (Agilent Technologies, Santa Clara, CA, USA).

### Library preparation and sequencing

Library preparation and sequencing was performed at Novogene (Wan Chai, Hong Kong). A total amount of 1.5 μg RNA per sample was used as input material for library preparations. Sequencing libraries were generated using NEBNext® Ultra RNA Library Prep Kit for Illumina® (NEB, Ipswich, MA, USA) following manufacturer’s recommendations. Briefly, mRNA was separated from total RNA using poly-T oligo-attached magnetic beads. Fragmentation was carried out using divalent cations under elevated temperature in NEBNext First Strand Synthesis Reaction Buffer (5 ×). First strand cDNA was synthesized using random hexamer primer. After second strand cDNA synthesis, remaining overhangs were blunted and NEBNext Adaptors with hairpin loop structure were ligated to prepare for hybridization. In order to select cDNA fragments of preferentially ca. 150–200 bp in length, the library of fragments was purified with AMPure XP system (Beckman Coulter, Beverly, MA, USA). Adapter-ligated fragments were amplified by PCR and the amplicons purified with the AMPure XP system. Library quality was assessed on the Agilent Bioanalyzer 2100 system on an Illumina Hiseq 2500 platform to generate paired-end reads.

### Data analysis

#### Quality control

The data quality control was performed with the CASAVA v.1.8 software. Clean reads were obtained by removing reads containing adapter, reads containing poly-N and low-quality reads from the raw data. At the same time, Q20, Q30, GC-content and sequence duplication level of the clean data were calculated. All downstream analyses were based on clean, high-quality data.

#### Transcriptome assembly

Raw reads were mapped to the genomes of tomato (*S. lycopersicum*) and pepper (*C. annuum*) using the software BBDuk (BBMap – Bushnell B. – https://sourceforge.net/projects/bbmap/) to detect and remove the sequences of these species ‘contaminating’ the pool of *A. swirskii* reads. The transcriptome was assembled ‘de novo’ using Trinity version r20140413p1 (Grabherr et al. 2011) with min_kmer_cov set to 2 and all other parameters were set at their default values. A hierarchical clustering of the contigs was performed with Corset v.1.05 (Davidson and Oshlack 2014). Moreover, the transcriptome sequences were inspected by the NCBI services for contamination by other species (e.g., human, plant tissue) through the Contamination Screen before releasing the final dataset. All the contaminating sequences found were removed. The number of possible allelic variants in the transcriptome released was assessed using the CD-HIT EST software (Huang et al. 2010).

#### Gene functional annotation

The gene function of the *A. swirskii* unigenes resulting from the assembly was annotated using the software NCBI blast (v.2.2.28 +) based on the following databases: Nr, Nt, KOG (EuKaryotic Orthologous Groups; Koonin et al. 2004) and UniProt/Swiss-Prot, and the description with the highest score calculated from the E-value was accepted. Also, functional annotation was performed using the software KAAS (Moriya et al. 2007) based on the KO database (KEGG Ortholog; Kanehisa et al. 2016) and the software hmmscan (HMMER 3, 2019; http://hmmer.org) against the Pfam database (Protein family; Finn et al. 2014) was run for the longest translated open reading frame of each unigene. Finally, the GO (Gene Ontology; The Gene Ontology Consortium 2019) annotation for each top BLAST hit was performed using BLAST2GO (Gotz et al. 2008).

#### Differential expression analysis

Gene expression levels were estimated by RSEM (Li and Dewey 2011) for each sample, using Bowtie2 aligner (v.2.3.0) and mismatch parameter 0. Differential expression analysis of the two conditions, exposure to tomato leaves or to pepper leaves, was performed using the DESeq R package v.1.10.1 (Anders and Huber 2010). The resulting P values were adjusted using Benjamini and Hochberg’s approach for controlling the false discovery rate. Genes with an adjusted P-value < 0.05 found by DESeq were assigned as differentially expressed.

#### Phylogenetic analysis of GSTs, P450s and CCEs

Sequences containing annotations referring to GST, P450 and CCE proteins were manually selected from the transcriptome based on their annotation. Unigenes with the same BLAST results and > 95% identity were considered redundant and only the largest contig was included in further analyses. All possible open reading frames (ORFs) were found using ORF finder (www.ncbi.nlm.nih.gov/orffinder/), then double-checked using BLASTP against the Nr database (https://blast.ncbi.nlm.nih.gov/Blast.cgi) and the hmmer (HMMER 3) to identify the Pfam homologous regions of each unigene. The nucleotide sequences were translated and the amino acid sequences aligned with Clustal W (v.2) (Larkin et al. 2007). *Tetranychus urticae, Galendromus* (= *Metaseiulus*) *occidentalis* (Nesbitt), *Neoseiulus barkeri* Hughes, *Apis mellifera* L. and *Drosophila melanogaster* Meigen protein sequences were collected from the available proteomes on NCBI (Tables S7–S9).

Phylogenetic trees of the CYP and GST protein families were determined by a maximum likelihood approach (RAxML) and bootstrapping with 1000 replicates. For these protein models the best fitting model was automatically determined by the program. Phylogenetic analysis of the CCEs using RaXML provided poor support of some of the tree clades causing ambiguities in the tree topology and a Bayesian inference using Mr. Bayes was selected, as more robust. The multiple sequence alignment of CCEs was trimmed at both ends according to the parameters previously set by Claudianos et al. (2006). The best substitution model for the protein alignment was found using the software MEGA v.4 (Tamura et al. 2007). According to the Akaike information criterion, the WAG + I + G + F model was selected for the phylogenetic analyses of CCEs. The Bayesian inference for the CCE family was employed with the following parameters: Metropolis-coupled Markov chain Monte Carlo sampling was performed with four chains and the heating parameter of 0.2. Starting trees were random and the analyses were performed for two runs of three million generations. Samplings were performed every 100 generations and the initial 25% of trees represented burn-in. The analyses were stopped when the average standard deviation of split frequencies dropped below 0.01.

## Results

### High-throughput sequencing, assembly and annotation

Eight cDNA libraries were constructed from RNA samples isolated from adult female mites exposed to either tomato or pepper leaves. The sequencing of the libraries resulted in a total of 893,330,940 clean reads of 150 base pairs (bp) long and 134.01 Giga base pairs (Gbp) – after the removal of adapters – reads containing poly-N, low-quality reads from the raw reads and the contamination from tomato and pepper (Table S1). This dataset was uploaded to the National Centre for Biotechnology Information (NCBI) with Sequence Read Archive (SRA) accession number PRJNA484730 and transcriptome shotgun assembly (TSA) accession numbers GHIT01000001-GHIT01070955. The sequencing quality of the clean reads was at the Q20 level for more than 96.6% of the clean reads and it was higher than Q30 for more than 91% of them, based on the base-calling quality scores of Illumina (Table S1). The average GC content across the eight samples was 48.3%. The clean reads were then assembled into 71,345 transcripts and 71,336 unigenes (Table S2). 71,345 sequences were screened for further contamination, of which 390 were removed, resulting in 70,955 sequences. The length distributions demonstrated that 77.5% of the transcripts and the unigenes (55,316 and 55,313, respectively) were between 500 and 2000 bp and that 24% of the transcripts and unigenes (17,090) were longer than 1000 bp (Figure S1). The mean length of transcripts and unigenes was 1441 bp (Table S2). The transcripts (70,955) were grouped into 56,262 clusters at 90% sequence identity, using the CD-HIT EST software. These results suggest that in our transcriptome, 14,693 of the transcripts (20.7%) are possible allelic variants.

After annotation, 47,159 (66.1%) of all unigenes successfully matched known genes in at least one of the seven databases used (Table S3). Overall, 10,573 unigenes were annotated in five of the databases (Figure S2). The E-value distribution of the annotated unigenes suggested that 68.2% of the mapped unigenes had very significant homology with the top hit (E-value < E^−45^); for the other 31.8% the values were lower, between E^−45^ and E^−5^, but still significant (Figure S3). The sequence identity with the top hit was at least 80% for more than half of the total annotated unigenes (58.6%) (Figure S4). As expected, *A. swirskii* is more similar to other arachnids than to species from other arthropod classes; 81.8% of the unigenes (12,033) had their best hits with sequences from *G. occidentalis* (a predatory mite), followed by those from the tick *Ixodes scapularis* Say (1.5%), the spider *Stegodyphus mimosarum* Pavesi (0.9%) and the aphid *Acyrthosiphon pisum* (Harris) (0.5%) (Figure S5).

According to the BLAST2GO results, 34,270 unigenes were classified into 196,539 GO terms and 56 subcategories (Figure S6). Also, in total, 23,070 unigenes were mapped and sorted into 26 KOG categories (Table S3, Figure S7). The KEGG pathway analysis resulted in a total of 15,927 unigenes (22.3%) mapped to 228 metabolic pathways (Table S3) and the 15 of those pathways, most abundant in unigenes, are presented in Figure S8.

### Differential expression analysis

**Fig. 1:**
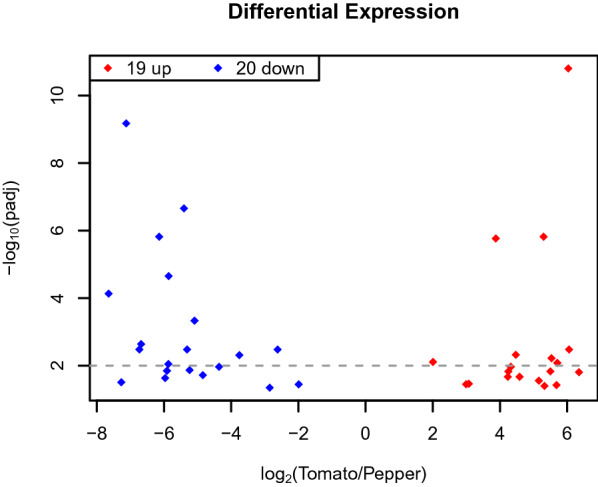
Volcano plot showing differentially expressed *Amblyseius swirskii* genes when exposed to tomato leaves compared to those when exposed to sweet pepper leaves (adjusted P < 0.05). Red dots represent upregulated genes, blue dots represent downregulated genes and the dashed line represents adjusted P < 0.01

**Fig. 2:**
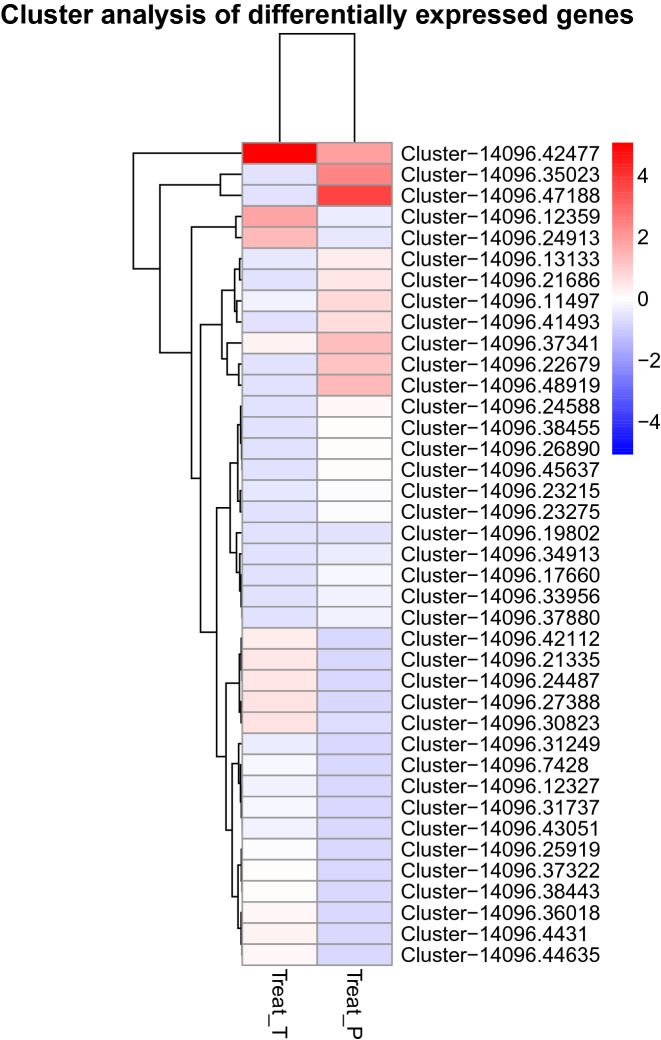
Hierarchical clustering heat map of 39 differentially expressed unigenes of *Amblyseius swirskii* mites exposed to tomato leaves (Treat_T) compared to mites exposed to pepper leaves (Treat_P) (adjusted P < 0.05). Colours from red to blue represent the fold change in gene expression from positive to negative, respectively

The analysis of differential gene expression resulted in 39 differentially expressed genes (DEGs) between mites exposed to tomato and those exposed to pepper leaves in four biological replicates (p_adj_ < 0.05), with expression differences > twofold (Fig. 1). Among these DEGs, 19 were up-regulated and 20 down-regulated in mites exposed to tomato leaves compared to mites exposed to pepper leaves (Figs. 1 and 2). Overall, 14 DEGs were found to be exclusively expressed in mites released on tomato leaves and 14 DEGs were expressed only in mites released on pepper leaves (Figs. 1 and 2).

After functional annotation, 25 out of the 39 DEGs were significantly homologous to previously annotated genes and nine DEGs were similar to uncharacterized protein sequences of *G. occidentalis* (Table 1). For the remaining five DEGs, it was not possible to find any significant homology to published sequences (Table 1).

**Table 1:** Summary of the annotated differentially expressed genes (DEGs)

Unigene_id	Nonredundant (Nr) description	Expression—exclusive
Cluster-14096.42112	Solute carrier family 22 member 7	Up—E
Cluster-14096.4431	Retinol dehydrogenase 12	Up—E
Cluster-14096.30823	Lipase member K	Up
Cluster-14096.25919	Mucolipin-3	Up—E
Cluster-14096.36018	Uncharacterized protein LOC100899406	Up
Cluster-14096.44635	No hit	Up
Cluster-14096.42477	No hit	Up
Cluster-14096.38443	Uncharacterized protein LOC100906407	Up
Cluster-14096.7428	Uncharacterized protein LOC100907771	Up
Cluster-14096.12327	Dual oxidase	Up—E
Cluster-14096.21335	Sodium-coupled monocarboxylate transporter 2	Up
Cluster-14096.27388	Neuropilin and tolloid protein 2, partial	Up—E
Cluster-14096.24487	No hit	Up
Cluster-14096.43051	Clavesin-2	Up—E
Cluster-14096.31249	Small G protein signaling modulator 1	Up—E
Cluster-14096.24913	Uncharacterized protein LOC100901357	Up
Cluster-14096.12359	Uncharacterized protein LOC100906988	Up
Cluster-14096.37322	Paired amphipathic helix protein Sin3a	Up—E
Cluster-14096.31737	N-acetylated-alpha-linked acidic dipeptidase 2	Up—E
Cluster-14096.41493	Lipase member K	Down—E
Cluster-14096.24588	E3 ubiquitin-protein ligase MIB1	Down—E
Cluster-14096.45637	Solute carrier family 22 member 7	Down—E
Cluster-14096.23275	Protein kinase C-binding protein 1	Down—E
Cluster-14096.22679	Uncharacterized protein LOC100901481	Down
Cluster-14096.33956	Protein PRRC1	Down—E
Cluster-14096.35023	DNAJ homolog subfamily C member 8	Down—E
Cluster-14096.11497	Uncharacterized protein LOC100902619	Down
Cluster-14096.17660	Methylcrotonoyl-CoA carboxylase beta chain, mitochondrial	Down—E
Cluster-14096.21686	Trifunctional purine biosynthetic protein adenosine-3	Down—E
Cluster-14096.13133	Sodium-dependent glucose transporter 1	Down
Cluster-14096.26890	Glycosyltransferase protein LARGE2	Down—E
Cluster-14096.34913	ATP-dependent RNA helicase DDX5	Down
Cluster-14096.48919	No hit	Down
Cluster-14096.38455	Mitochondrial carrier homolog 2	Down—E
Cluster-14096.19802	Adenylate cyclase type 6	Down—E
Cluster-14096.37880	Uncharacterized protein LOC100901512	Down
Cluster-14096.47188	No hit	Down
Cluster-14096.37341	Uncharacterized protein LOC100897466	Down
Cluster-14096.23215	Histone-lysine N-methyltransferase, H3 lysine-79 specific	Down

Up- or down-regulation of gene expression (up/down) in mites exposed to tomato leaves (Treat_T) compared to mites exposed to pepper leaves (Treat_P). Exclusive (E) indicates exclusive gene expression in mites exposed to either tomato or pepper leaves

Among the 19 DEGs up-regulated in mites exposed to tomato leaves, 11 had annotated homologues in public databases (Table 1). Of those, two DEGs showed higher expression levels in mites exposed to tomato leaves. The two DEGs were significantly similar to a *G. occidentalis* lipase member K; one being a protein involved in lipid degradation and metabolism and the other a sodium-coupled monocarboxylate transporter 2 (Table 1). The other nine annotated DEGs were expressed in mites exposed to tomato leaves but not expressed in mites exposed to pepper leaves; they were homologous to the *G. occidentalis* proteins as described in Table 1.

Moreover, 14 of the 20 down-regulated DEGs in mites exposed to tomato leaves had significant homologies to previously annotated proteins (Table 1). Three of those had lower expression levels in mites exposed to tomato compared to mites exposed to pepper and were homologous to the following *G. occidentalis* proteins: ATP-dependent RNA helicase DDX5, sodium-dependent glucose transporter 1 and histone-lysine N-methyltransferase (Table 1). The other 11 DEGs were expressed in mites exposed to pepper leaves and not in mites exposed to tomato leaves; they had a significant homology to previously described *G. occidentalis* proteins (Table 1).

### Detoxification superfamilies CYPs, GSTs and CCEs

**Fig. 3:**
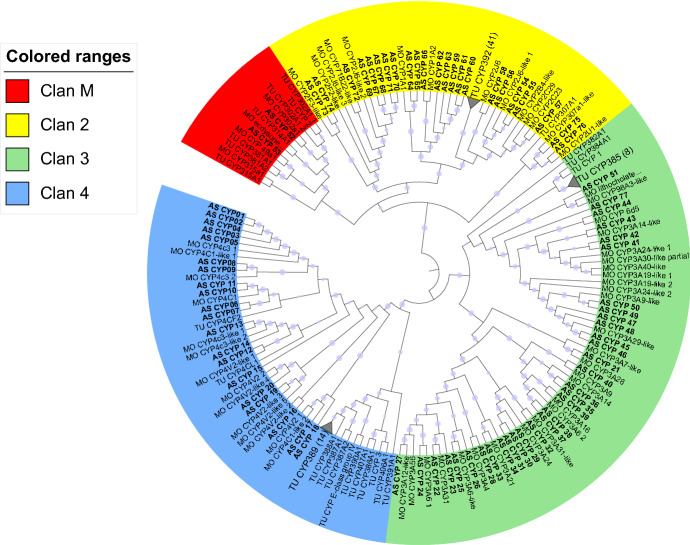
Phylogenetic analysis of 77 *Amblyseius swirskii* (AS) cytochrome P450 (CYP) proteins represented with those of *Tetranychus urticae* (TU) and *Galendromus* (*Metaseiulus*) *occidentalis* (MO). The CYP protein clans M (mitochondrial), 2, 3 and 4 are represented in four colours. The midpoint-rooted tree was generated using a maximum likelihood approach (RAxML v.8.2.10) and bootstrapping with 1000 replicates. Blue dots on the nodes represent bootstrap likelihood > 0.5; their size represents their relative value

In the *A. swirskii* transcriptome, 162 unigenes coding for P450s from the Nr annotation were identified. After manually removing those with short ORFs (< 400 bp long), or with > 95% identity, along with the published P450s of *T. urticae* and *G. occidentalis* (Grbić et al. 2011; Wu and Hoy 2016) the remaining 77 P450 unigenes were used to construct a phylogenetic tree, (Fig. 3, Table S4). Cytochrome P450s were classified into four major families; as indicated by the closest hits on the NCBI Nr database and the phylogenetic tree (Fig. 3, Table S4) our analysis illustrated 24 unigenes from *A. swirskii* belong to the CYP2 family, 31 to CYP3, 20 to CYP4 and two to mitochondrial P450s.

**Fig. 4:**
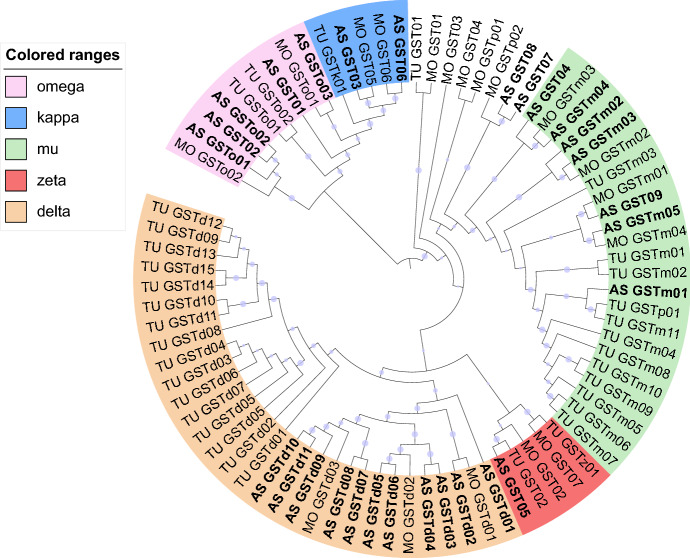
Phylogenetic analysis of 28 cytosolic *Amblyseius swirskii* (AS) glutathione S-transferase (GST) proteins represented with those of *Tetranychus urticae* (TU) and *Galendromus* (*Metaseiulus*) *occidentalis* (MO). The GST protein classes Omega, Kappa, Mu, Zeta and Delta are represented in five colours. Non-coloured proteins belong to uncharacterized clades. The midpoint-rooted tree was generated using a maximum likelihood approach (RAxML v.8.2.10) and bootstrapping with 1000 replicates. Blue dots on the nodes represent bootstrap likelihood > 0.5; their size represents their relative value

In addition, 81 GST genes were identified in the *A. swirskii* transcriptome and, after filtering for short open reading frames (< 200 bp long) and high sequence similarity (> 95%), 28 putative GST genes were selected to be used in the phylogenetic analysis along with previously published *T. urticae* and *G. occidentalis* GST proteins (Fig. 4). Based on the phylogenetic analysis and on the annotations from the NCBI Nr database (Fig. 4, Table S5), these GST-related unigenes were assigned to the GST classes as follows; 11 to the Delta/Epsilon, seven to the Mu, one to the Zeta, five to the Omega, two to the Kappa, and three to other classes of GSTs.

**Fig. 5:**
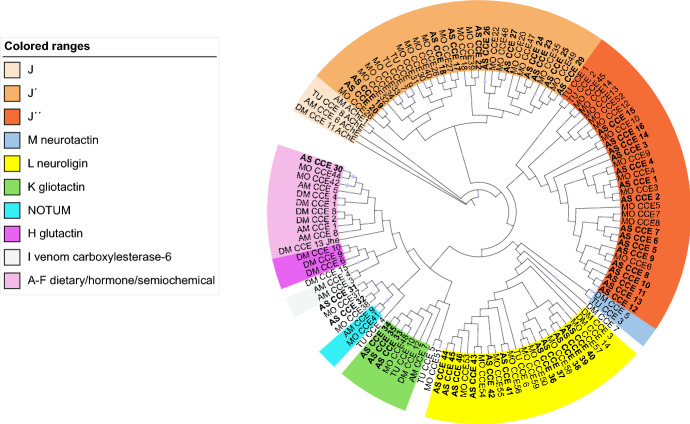
Phylogenetic analysis of 44 *Amblyseius swirskii* (AS) amino acid sequences of carboxyl/cholinesterase (CCE) genes, represented with those of *Tetranychus urticae* (TU) and *Galendromus* (*Metaseiulus*) *occidentalis* (MO). The various CCE classes/clades J, J’, J”, K, L, M, H, A–F, NOTUM are represented in different colours. The alignment was trimmed at the N- and C-terminal (Claudianos et al. 2006). The midpoint-rooted tree was generated using Mr. Bayes XSEDE. Blue dots on the nodes represent posterior probabilities > 0.5; their size represents their relative value

After manual identification of 96 annotated CCEs in the transcriptome of *A.* s*wirskii,* 44 nonredundant, putative full-length ORFs were selected. The translated CCE protein sequences were assigned to the following clades: 11 to the J’, 16 to the J”, 3 to the K, 11 to the L and two to uncharacterized clades, according to the phylogenetic analysis known for *T urticae* and *G. occidentalis* CCEs (Fig. 5, Table S6). The majority of *A. swirskii* CCEs fall into clades J’ and J” which are described as Acari-specific clades (Wu and Hoy 2016) (Fig. 5, Table S6).

## Discussion

A deep analysis of *A. swirskii* transcriptomic response to the tomato leaves in comparison with their response to pepper, a favourable host plant, was performed. A significant amount of sequencing data was generated (134.01 Gbp) in our RNAseq approach which were used to assemble and annotate a transcriptome, resulting in a total of 47,159 annotated unigenes. This large transcriptome is a significant genomic resource, now available to the scientific community, from a species with very limited public genomic information.

It has been estimated that 20.7% of the total transcriptome sequences are possible allelic variants. However, a firm conclusion regarding the number of allelic variants cannot be drawn, due to the lack of available annotated genome of *A. swirskii*. Different variants in the population, alternative splicing, or errors in the alignments of the transcripts can be counted as different unigenes erroneously if the transcriptome complexity is high (Chang et al. 2014). Moreover, given the nature of the experiment and the biology of the mite, the raw data generated contained contamination from tomato, pepper and the *C. edulis* pollen food source. Tomato and pepper sequences were filtered and removed from the transcriptome, because there is enough genomic information in the databases for these sources of contamination. However, in the case of contamination with *C. edulis* transcripts, there are no genomic resources available to properly remove them, so it is possible that they contribute to the high number of unigenes in the transcriptome. We decided to perform a 48-h exposure time with starvation in order to minimize the contamination by the food source mRNA in the transcriptome, as it has been shown for another phytoseiid that 24-h starvation time was not sufficient to eliminate the detection of prey sequences (Hoy et al. 2012). Moreover, even though the genome of *T. urticae* is available, we decided not to provide *T. urticae* as prey to the predators, because highly conserved sequences can cause chimeric assemblies and transcript fragmentation. Nevertheless, our experimental design guaranteed that the mites were under identical conditions during the treatments. Therefore, the differential expression measured was related to the response of the mite to tomato and to pepper.

Our analyses elucidated a differential transcriptomic response of *A. swirskii* transferred from pepper to tomato for 48 h, involving 39 genes. It has been shown that when *T. urticae* is transferred from bean to tomato leaves for 12 h, the expression of more than 400 genes is altered (Dermauw et al. 2013). Herbivorous mites consuming plant tissue are more exposed to the plant allelochemicals than their predators and have adapted to detoxify/excrete these toxins. Their predators, on the other hand, are exposed to a smaller spectrum of the plant toxins, either indirectly through their ingested prey or by contact with the toxins excreted on the plant surface. The low number of DEGs found in this study, support the notion that the predatory mites are less adapted to detoxify plant toxins compared to their herbivorous prey.

Among the differentially expressed genes there are five transcripts encoding transmembrane transporters (GO ID: 0,055,085), three of which belong to the Major Facilitator Superfamily (MFS) (PFAM ID: PF07690). Members of the MFS were up-regulated in spider mites transferred from bean to tomato for 12 h as well (Dermauw et al. 2013). MFS transporters are responsible for transporting metabolites out of the cell and contribute to cellular detoxification processes (Feyereisen et al. 2015; Van Leeuwen & Dermauw 2016).

Moreover, we found two annotated transcripts: one alcohol dehydrogenase and one carboxyl transferase possibly related to the metabolism of the most abundant tomato exudates, the acyl sugars. These are polyols with multiple hydroxyl groups and consist of aliphatic acyl groups esterified to sucrose (Schilmiller et al. 2010); hence, they are putative substrates for arthropod alcohol dehydrogenases (McKechnie and Geer, 1984). Acyl sugars accumulated on the bodies of the predatory mites after walking on tomato stems and are highly toxic to them (Paspati et al. 2021). The sticky acyl sugars can cover the cuticle and either block the mite respiratory spiracles causing their suffocation or penetrate and degrade the underlying cellular membranes causing their desiccation (Puterka et al. 2003). A chitin synthesiser (Cluster-14096.7428) which synthesizes the major component of the cuticle was found to be upregulated in mites exposed to tomato. This chitin synthesiser is of great interest because it might induce the thickening of the cuticle, as a response against the tomato acyl sugars, that destroy the cellular membranes under the cuticle. A chitin synthesiser has been suggested to play an important role in mite resistance to acaricides in the phytophagous mite *Panonychus citri* (McGregor) (Niu et al. 2012). Cuticle thickening has also been shown to be associated with insecticide resistance in insects (Wood et al., 2010). The penetration of insecticides has been found to be affected by thickened cuticles, as well as by other components of the cuticles, such as surface hydrocarbons (Pedrini et al., 2009). Two other transcripts (Cluster-14096.12359, Cluster-14096.37322) involved in oxidations reduction (GO: 0,055,114) might be expressed as a response to trichome oxidases such as the polyphenol oxidases, which are involved in the production of quinones, substances harmful to arthropods (Glas et al. 2012).

Intriguingly, none of the 39 differentially expressed genes detected in this study belong to known families of detoxification enzymes such as cytochrome P450s, GSTs and CCEs. This is in line with the results from a recent study that found a weak transcriptomic response to synthetic and natural acaricides in *Phytoseiulus persimilis* Athias-Henriot predatory mites (Bajda et al., 2022). A differential gene expression analysis carried out in this species detected only two transcripts differentially expressed in mites treated with fenbutatin oxide, and 34 transcripts in orange oil-treated mites, of which only one encoded a classical detoxification gene (Bajda et al. 2022). These results suggest that Phytoseiidae mites are most likely characterized by mild transcriptomic responses to xenobiotics. Moreover, the fitness of another predatory mite, *Neoseiulus californicus* (McGregor), does not increase over many generations, when reared on an ‘unfavourable’ hosts such as tomato (Cédola et al. 2001). In contrast, the adaptation of the herbivorous mite *T. urticae* to tomato involved a strong transcriptional response that included genes from detoxification families and the fitness of spider mites increased rapidly on tomato (Dermauw et al. 2013; Wybouw et al. 2015). Most likely, the predators are not characterized by the fine-tuned evolutionary adaptation of the polyphagous herbivores to phytotoxins, because they are exposed to less phytotoxins through their diet.

In this study we have described a significant number of genes belonging to three families of detoxification enzymes. Among monooxygenases cytochrome P450 (CYP), 77 genes were identified (Fig. 4, Table S3). This number is similar to that recently described in other acarine species such as *G. occidentalis* (n = 63) (Dermauw et al. 2020; Wu and Hoy 2016) or *T. urticae* (n = 78) (Dermauw et al. 2020; Grbić et al. 2011). In other arthropods the number of CYPs ranged from 43 in the predatory mite *N. barkeri* (Cong et al. 2016) to 121 in the spider mite *P. citri* (Niu et al. 2012) with a significant variation among arthropod species (Zimmer et al. 2014). This family of enzymes plays a crucial role in the detoxification pathways of xenobiotic compounds such as plant toxins, secondary metabolites and pesticides (Scott et al. 1998; Schuler 2011) and they have been frequently associated with the evolutionary adaptation of several arthropod species to host plant metabolites and/or the development of resistance to pesticides (Yang et al. 2011; Dermauw et al. 2013; Niu et al. 2012). In arthropods, genes of this family fall into four major clades: CYP2, CYP3, CYP4 and the mitochondrial clade (Feyereisen, 2012). Clade CYP2 includes enzymes involved in breaking down pesticides and associated with the development of resistance in herbivorous populations (Grbić et al. 2011). Our analysis showed that *A. swirskii* and two other phytoseiids, *G. occidentalis* and *N. barkeri*, have fewer genes belonging to clade CYP2 compared to *T. urticae* (24, 16, 9 and 38, respectively) (Table 2; Dermauw et al. 2020; Wu and Hoy 2016; Cong et al. 2016; Grbić et al. 2011). However, among the three phytoseiids, *A. swirskii* has the most of CYP2 genes. Interestingly, in clade CYP3 the phytoseiids *A. swirskii, G. occidentalis* and *N. barkeri* (31, 23 and 19, respectively) have more genes compared to the spider mites *T. urticae* and *P. citri* (9 and 9, respectively; Dermauw et al. 2020; Wu and Hoy 2016; Cong et al. 2016; Grbić et al. 2011; Niu et al. 2012). Many enzymes from this clade have been associated with the resistance to a broad range of pesticides and also with the detoxification of host plant secondary metabolites (Dermauw et al. 2013). The number of members of the clade CYP4 is similar among phytoseiids and herbivorous mites: *A. swirskii* has 20 unigenes, *G. occidentalis* 19, *T. urticae* 26 and *P. citri* 26 (Dermauw et al. 2020; Grbić et al. 2011; Niu et al. 2012; Wu and Hoy 2016). However, in *N. barkeri* only two genes have been found to belong in this clade (Cong et al. 2016). Finally, the mitochondrial clade is the least represented in *A. swirskii* similar to other closely related phytoseiids such as *G. occidentalis* and *N. barkeri*, and the herbivores *T. urticae* and *P. citri* (Grbić et al. 2011; Niu et al. 2012; Cong et al. 2016; Wu and Hoy 2016).

**Table 2:** A comparison of cytochrome P450 (CYP), glutathione S-transferase (GST) and carboxyl/cholinesterase (CCE) gene numbers in the genomes of six acarine species, *Ixodes scapularis*, *Tetranychus urticae*, *Panonychus citri*, *Galendromus occidentalis*, *Neoseiulus barkeri* and *Amblyseius swirskii*

			*I. scapularis*	*T. urticae*	*P. citri*	*G. occidentalis*	*N. barkeri*	*A. swirskii*
CYP		CYP2		38	12	16	9	24
		CYP3		9	9	23	19	31
		CYP4		26	26	19	2	20
		Μitochondrial CYP		5	7	5	2	2
		Total		78	79	63	32	77
GST		Delta/Epsilon	12	16	7	3	3	11
		Mu	14	12	10	5	4	7
		Omega	3	2	1	3	1	5
		Sigma	0	0	2	0	0	0
		Theta	0	0	1	0	0	0
		Zeta	3	1	1	1		1
		kappa			2		2	2
		Unknown	0	0		1		3
		Total	32	31	24	13	10	28
CCE	Dietary/detoxification/hormone/semiochemical class	Clade A, B, C, D, E, F		0	7	2		1
	Neuro/developmental class	Clade H (glutactin)		0		0		0
		Clade I (NOTUM)		0		0		0
		Clade J (AChEs)		1		1		0
		Clade K (gliotactin)		2		2		3
		Clade L (neuroligins)		5		7		11
		Clade M (neurotactins)		1		0		0
	Acari-specific class	Clade J´		34		19		11
		Clade J´´		22		15		16
		Undetermined		3	5	3		2
		Total		71	13	44		44

Data derived from: *I. scapularis*, Reddy et al. (2011), *T. urticae*, Grbić et al. (2011), *P. citri*, Niu et al. (2012), *G. occidentalis*, Wu and Hoy (2016), *N. barkeri*, Cong et al. (2016), *A. swirskii* from the current study

Glutathione-S-transferases also play important roles in detoxification of xenobiotics and they were identified as well. GSTs are classified into Delta, Epsilon, Omega, Sigma, Zeta, and Theta classes (Fang 2012); among these classes, Delta, Epsilon and Mu are the largest in gene numbers in Acari (Table 2). Five classes of GSTs were found in *A. swirskii*, including Delta/Epsilon, Mu, Omega, Zeta and Kappa (Fig. 5, Table S4). The Delta/Epsilon class genes in *A. swirskii* are the most numerous (11); homologues of this class are also found in the acarines *G. occidentalis* (Wu and Hoy 2016) and *T. urticae* (Grbić et al. 2011). In arthropods, both insects and mites, the Delta/Epsilon class is involved in pesticide resistance (Chen et al. 2003; Pavlidi et al. 2015). The Mu class GST genes are homologous to mammalian Mu GSTs and have been identified in *A. swirskii* (7) and in other acarine species, such as *G. occidentalis* (Wu and Hoy 2016), *N. barkeri* (Cong et al. 2016), *I. scapularis* (Niranjan Reddy et al. 2011), *T. urticae* (Grbić et al. 2011) and *P. citri* (Niu et al. 2012). In general, the numbers of the Mu GSTs in all acarine species are similar to those of the Delta/Epsilon class. The Mu GSTs have been suggested to participate in pesticide resistance in *T. urticae* mites (Pavlidi et al. 2015). Five Omega GSTs were found in *A. swirskii*, more than found in *G. occidentalis* and other acarine (Wu and Hoy 2016). Omega GSTs are involved in the removal of S-thiol groups from proteins and, like Zeta GSTs, may also be involved in oxidative stress responses (Board et al. 2000; Meng et al. 2014). A single gene of the Zeta GSTs was identified in *A. swirskii* which is similar to other acarine species with the exception of *I. scapularis* which has three (Wu and Hoy 2016). These enzymes are involved in the catabolism of amino acids and possibly in pesticide resistance (Board et al. 1997). Two Kappa GSTs were identified in the *A. swirskii* transcriptome which is similar to *N. barkeri* (Cong et al. 2016). However, no Sigma, nor Theta, GST class genes were found in this species, as in *N. barkeri*, *G. occidentalis*, *I. scapularis* and *T. urticae* (Grbić et al. 2011; Niranjan Reddy et al. 2011; Cong et al. 2016; Wu and Hoy 2016), suggesting different composition of GSTs between acarines and insects.

Carboxyl/cholinesterases are also key enzymes associated with resistance to insecticides/acaricides and plant toxins in arthropods (Liang et al. 2007; Dermauw et al. 2013; Zhang et al. 2013). According to Claudianos et al. (2006), insect CCEs fall into three main phylogenetic classes with distinct functions: dietary/detoxification (A–C clades), pheromone/hormone processing (D–G clades) and neuro/developmental (H–M clades). In the transcriptome of *A. swirskii*, 44 CCEs and their phylogenetic relationships with those from other Acari species were investigated (Fig. 5, Table S5). The distribution of *A. swirskii* CCEs among CCE classes/clades was similar to that of *G. occidentalis* and *T. urticae*. First, *A. swirskii* CCEs, similar to *G. occidentalis*, has one homolog to insect CCEs in the dietary/detoxification and hormone/semiochemical classes (clades A–G) (Grbić et al. 2011; Wu and Hoy 2016). Secondly, most *A. swirskii* are homologous to the *G. occidentalis* CCEs which fall into two clades, J’ and J” (11 and 16, respectively) (Wu and Hoy 2016). This distribution pattern is similar to that of *M. occidentalis* and *T. urticae* CCE superfamily, supporting the hypothesis that the J’ and J” clades represent Acari-specific clades (Grbić et al. 2011; Wu and Hoy 2016). Some of the numerous mite CCEs in the J’ and J” clades are most likely involved in the detoxification of xenobiotics (e.g., insecticides and plant toxins), as genes of those classes were upregulated in acaricide-resistant strains of *T. urticae* and *P. citri* (Niu et al. 2012; Dermauw et al. 2013). In the neuro/developmental class, *A. swirskii* contains three orthologs of the clade K gliotactin, and 11 orthologs of CCEs were found in the clade L of neuroligins.

Our analysis showed that the abundance of genes in the CYP and CCE detoxification families is species-specific and differs between predatory and herbivorous mites. As described before, herbivorous mites are exposed to phytotoxins by ingestion and contact, whereas phytoseiid mites are exposed to a smaller range of toxins, indirectly by the ingested prey or directly by contact with excreted toxins. These differences in the range of and the route of exposure to plant toxins between phytoseiids and herbivorous mites may explain the differences in their detoxification machineries and the ability of herbivores to adapt to a poor-quality host, such as tomato, contrary to their predators (Grbić et al. 2011; Dermauw et al. 2013; Wybouw et al. 2015).

The implementation of IPM has become a priority for the EU since it is a scientifically based approach to pest control, with a strong drive to protect the environment from the negative impacts of conventional pesticides (Abrol 2013). Biological control agents are key for the success of IPM; they are major components of this strategy and contribute effectively to pest control, sometimes without a single pesticide input throughout the season (Abrol 2013). In this context, the predatory mite *A. swirskii* is a valuable player. We have created a thorough transcriptome database of *A. swirskii* and also identified the genes involved in its response to tomato, an unfavourable host plant. In addition, we have illustrated that phytoseiid mites have evolved a detoxification system similar to that found in their prey, but with key differences, which might explain their lack of success in adapting to the toxic tomato environment. Overall, our data will facilitate research on adaptive evolution of predatory mites to plant toxins and may serve as invaluable public data for other gene analysis of *A. swirskii*, an important biological control agent.

## Supplementary Information

Below is the link to the electronic supplementary material.

Supplementary file1 (DOCX 1001 kb)

Supplementary file1 (FASTA 96 kb)

Supplementary file1 (FASTA 174 kb)

Supplementary file1 (FASTA 45 kb)

Supplementary file1 (FASTA 130 kb)
