# Supplementary material for: Transcriptomic profile of the predatory mite Amblyseius swirskii (Acari: Phytoseiidae) on different host plants
Source: Exp Appl Acarol. 2022 May 9;86(4):479–98. doi: 10.1007/s10493-022-00715-w (PMC9110503; doi:10.1007/s10493-022-00715-w)
Supplement: Supplementary file 1 — Supplementary file1 (DOCX 1001 kb) [file 10493_2022_715_MOESM1_ESM.docx]

Journal of Experimental and Applied Acarology

Title: **The response of the predatory mite *Amblyseius swirskii* to tomato exudates**

Angeliki Paspati^1*^, Alberto Urbaneja, Joel González-Cabrera^2^

^1^ Instituto Valenciano de Investigaciones Agrarias (IVIA). Centro de Protección Vegetal y Biotecnología. Unidad Mixta Gestión Biotecnológica de Plagas UV-IVIA. Carretera Moncada-Náquera km 4,5. 46113 Moncada, Valencia, Spain

^2^ Universitat de València, Department of Genetics, Estructura de Recerca Interdisciplinar en Biotecnología i Biomedicina (ERI-BIOTECMED). Unidad Mixta Gestión Biotecnológica de Plagas UV-IVIA. Dr Moliner 50, 46100. Burjassot, Valencia, Spain.

^*^Current addresss: HAO-DEMETER, Institute of Olive, Subtropical crops and Viticulture, IOSV, Heraklion, Greece

Corresponding author: [joel.gonzalez@uv.es](mailto:joel.gonzalez@uv.es)

**Figures**


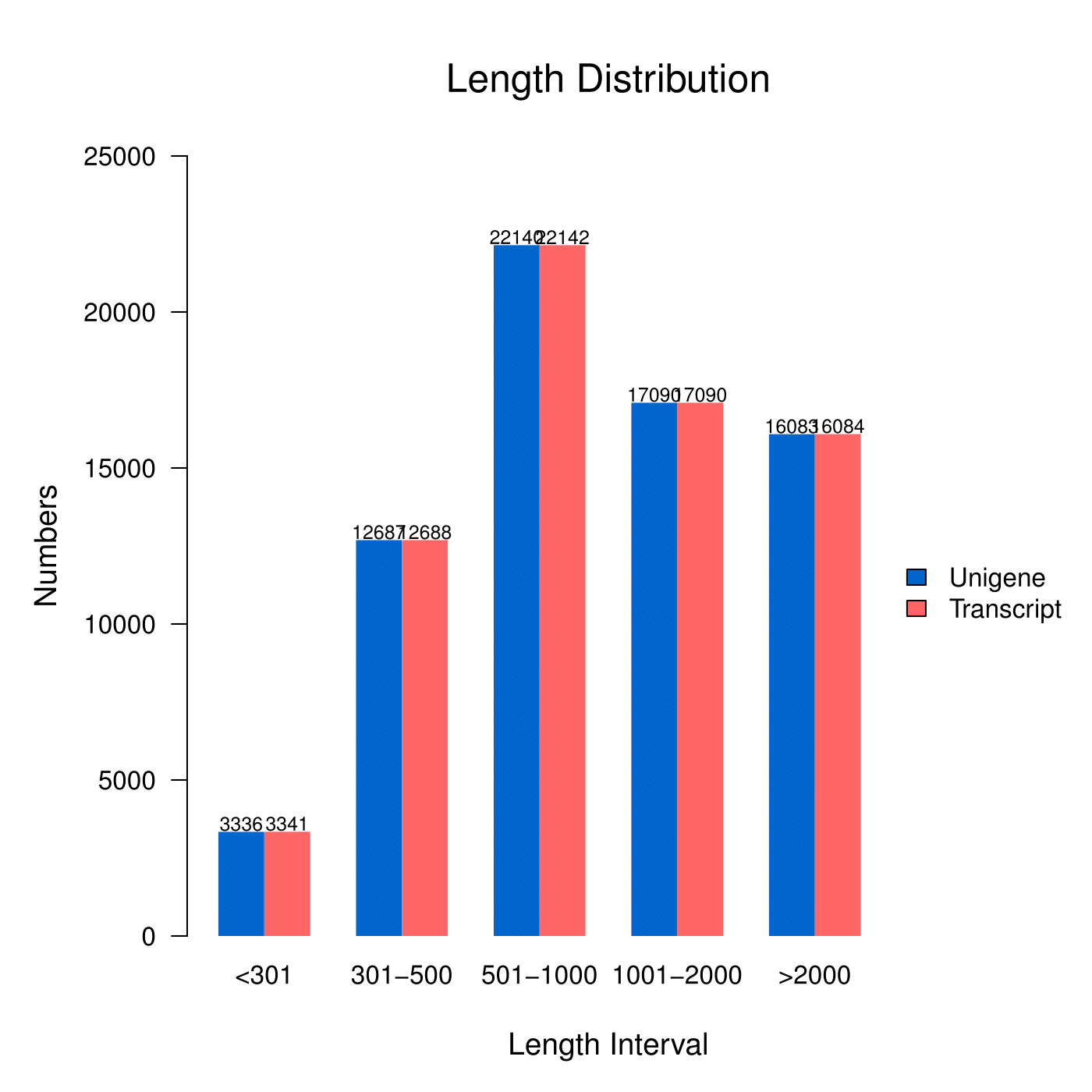


Figure S1. Length distribution of the assembled transcripts and unigenes obtained from the combined reads of eight transcriptome libraries of A. swirskii.


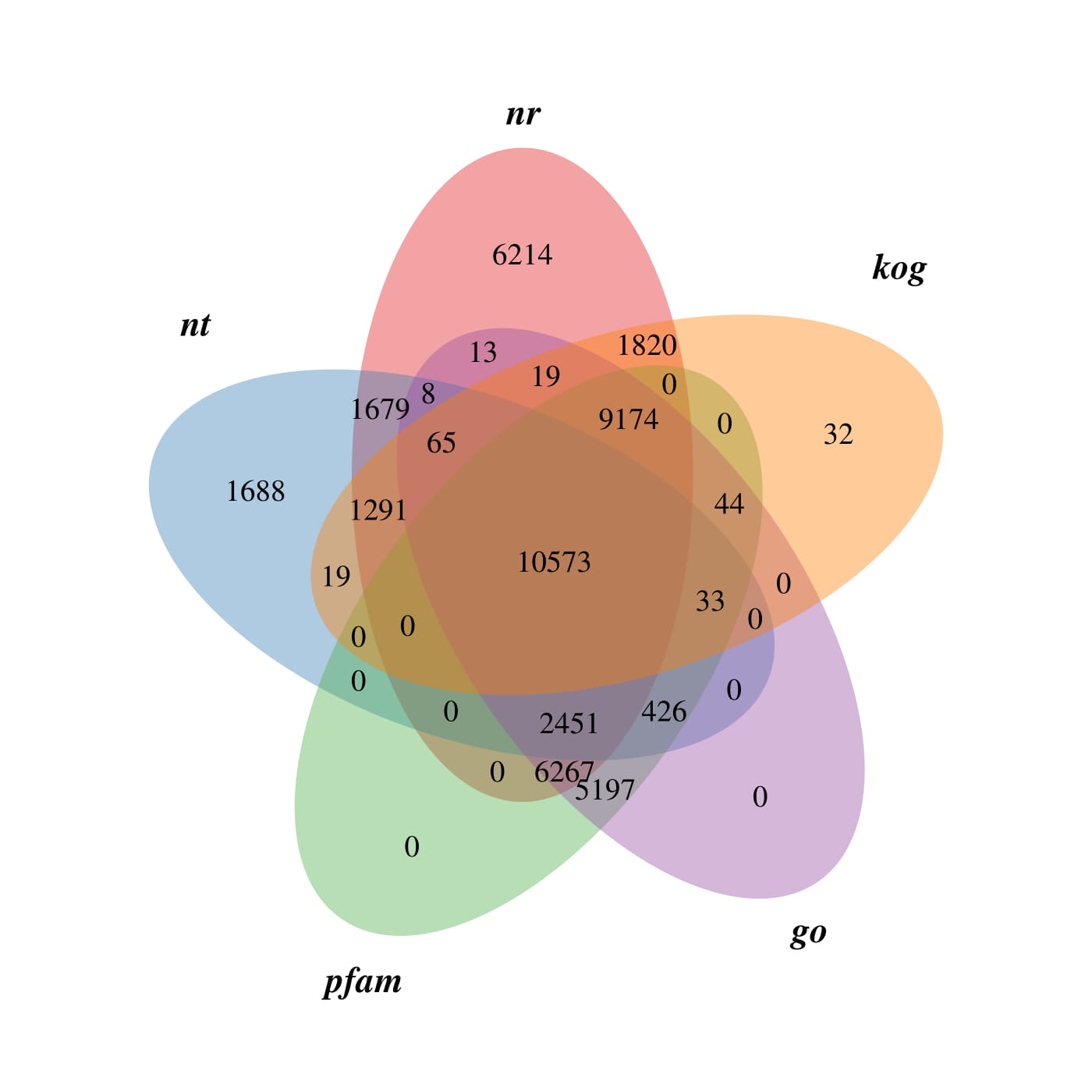


**Figure S2.** Venn diagram of unigenes of A. swirskii annotated using various databases. The Venn diagram shows the overlapping unigenes annotated in the Nr, Nt, pfam, GO and KOG databases.


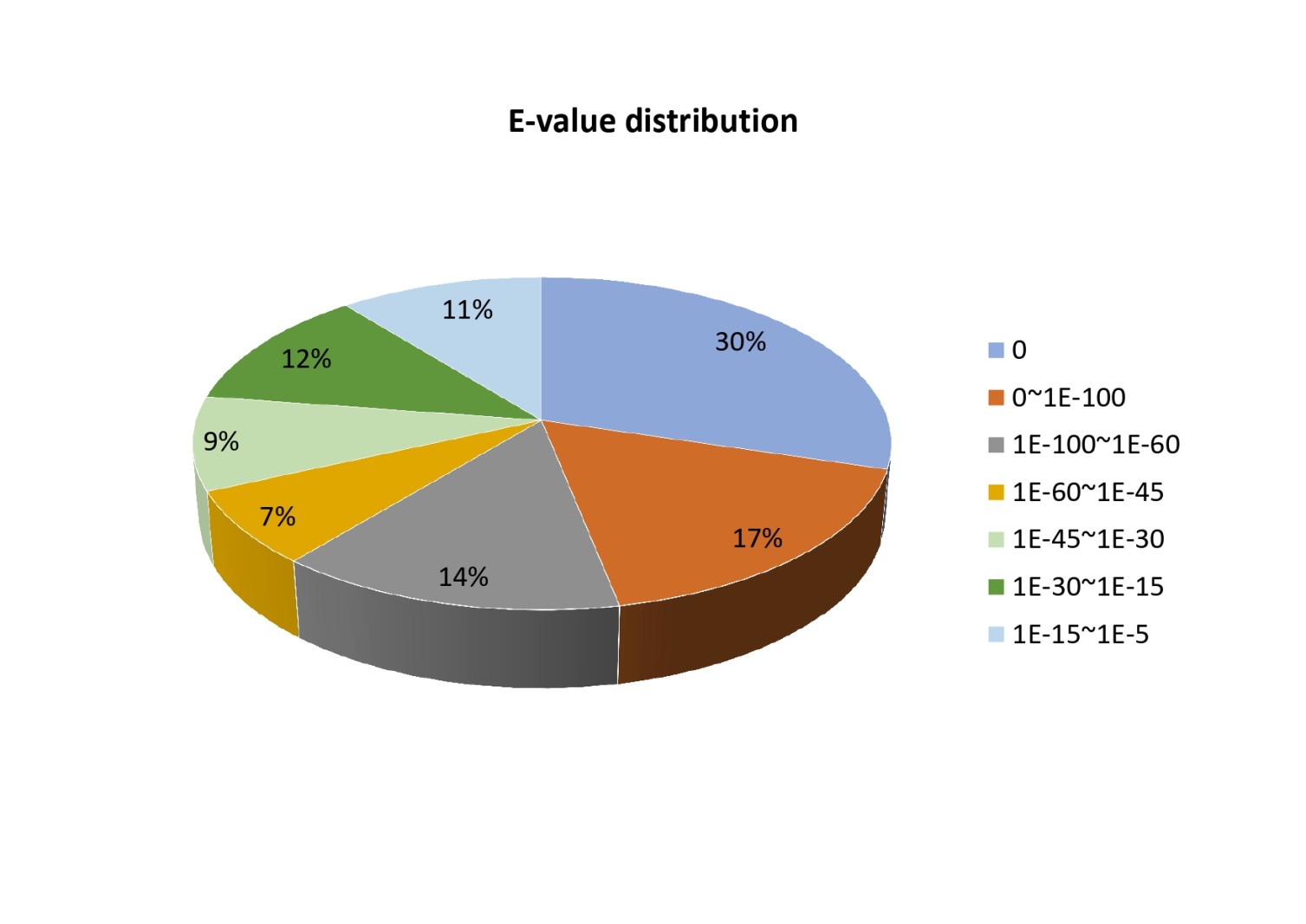


**Figure S3.** E-value distribution of the blast hits for each unigene with E-value ≤10^−5^ of A. swirskii transcriptome data.


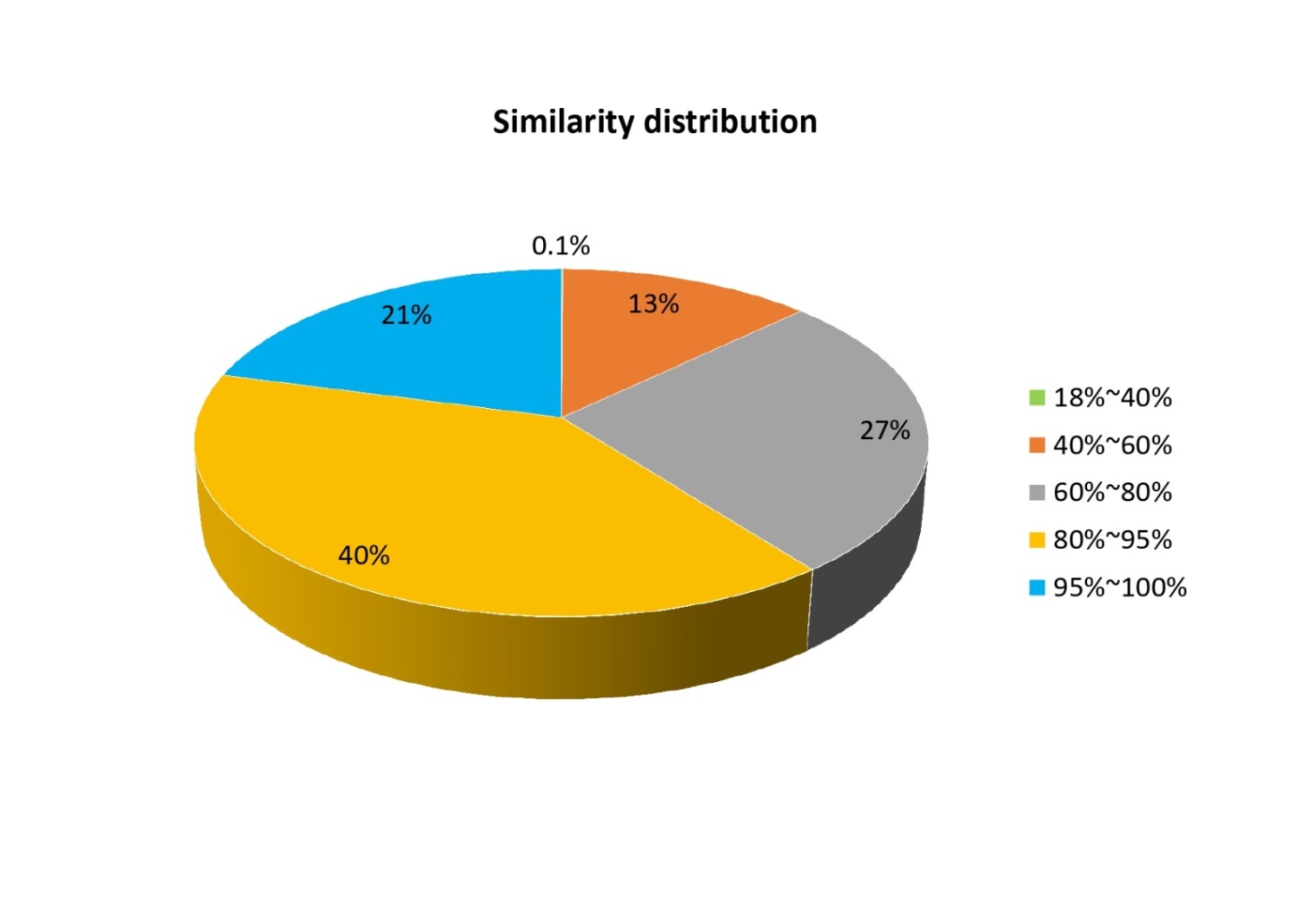


**Figure S4.** Similarity distribution of the top blast hits for each unigene sequence of A. swirskii transcriptome.


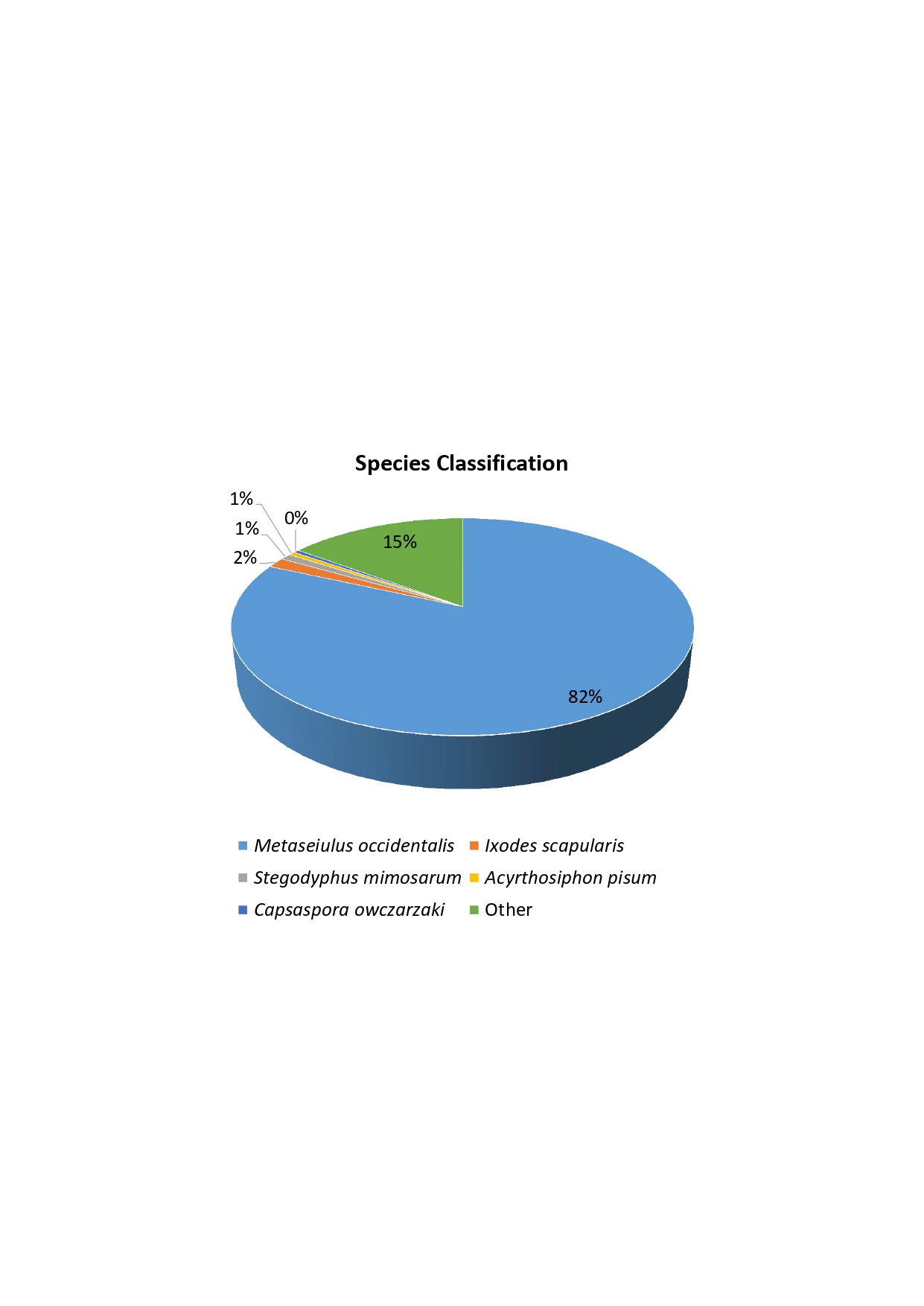


**Figure S5.** Species distribution of the total homologous sequences with E value ≤10^−5^. The first hit of each sequence was used for statistical analysis.


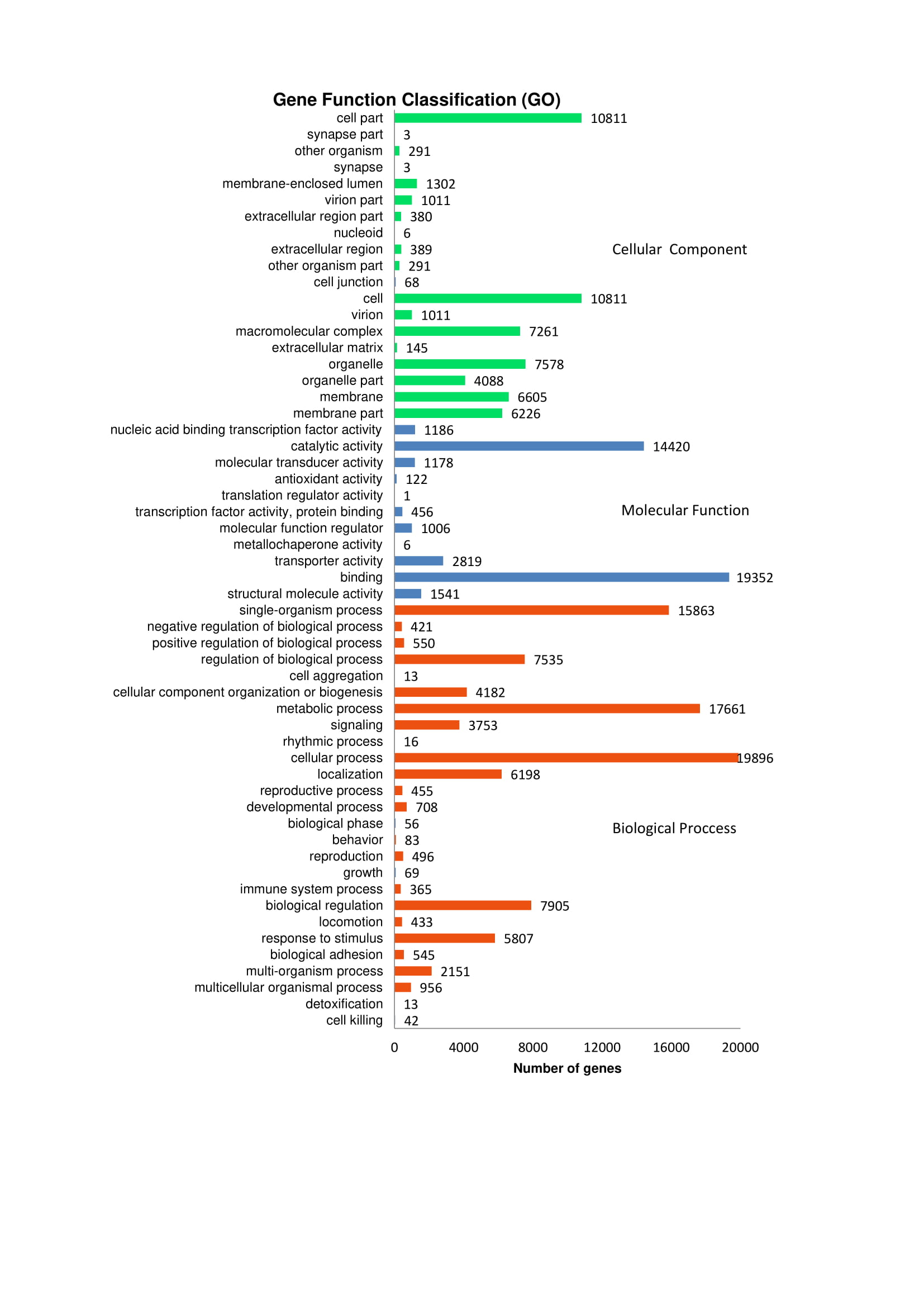


**Figure S6.** GO annotation of the overall unigene dataset. The total transcriptome dataset of A. swirskii was classified into biological process, cellular component, and molecular function subcategories. X-axis is the names of the 56 subcategories; Y-axis is the number of unigenes annotated under this subcategory.


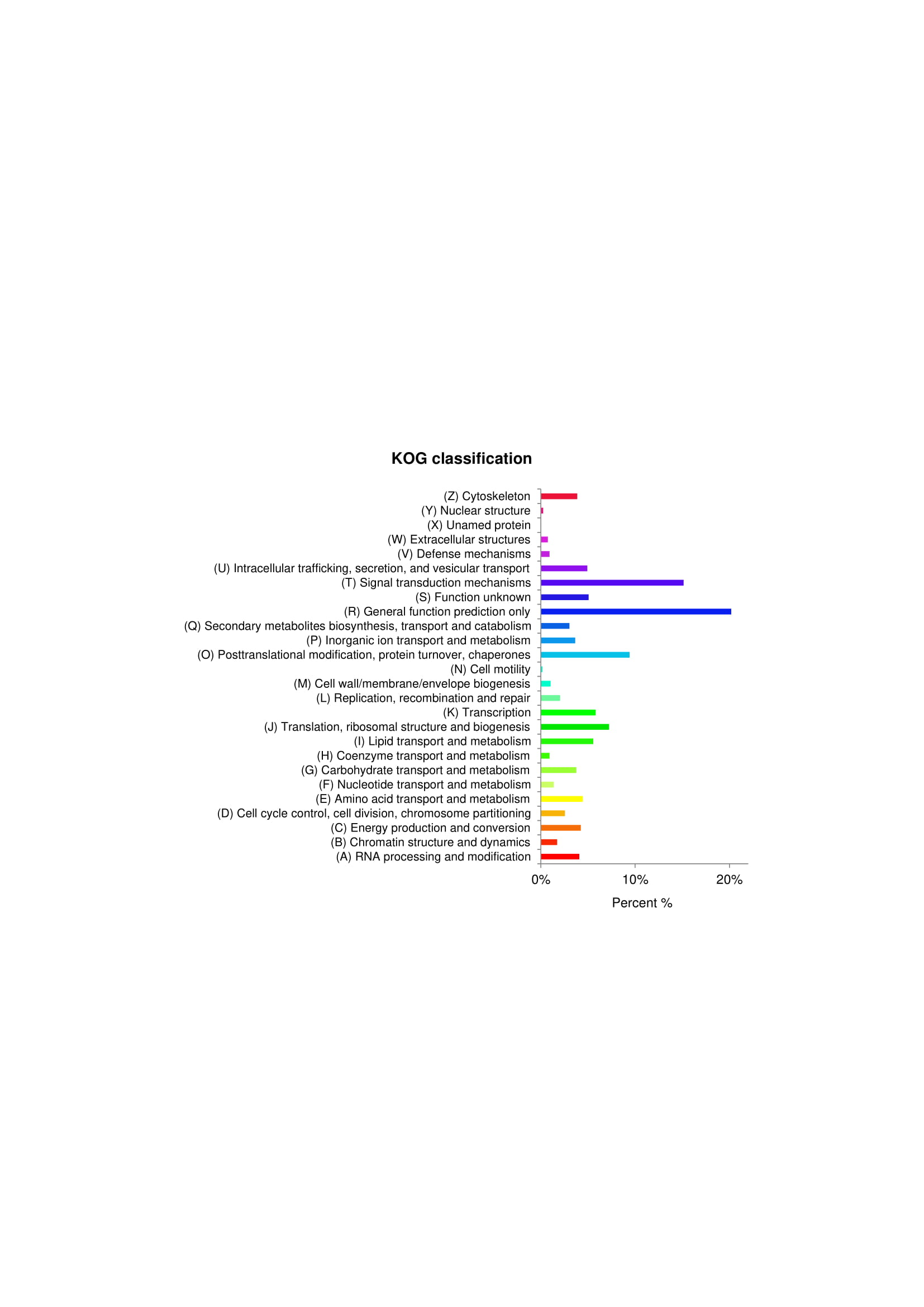


**Figure S7.** Classification of the euKaryotic Orthologous Groups (KOG) analysis of the A. swirskii transcriptome. X-axis is the names of the 26 KOG group; Y-axis is the percentage of genes annotated under this group in the total annotated genes.


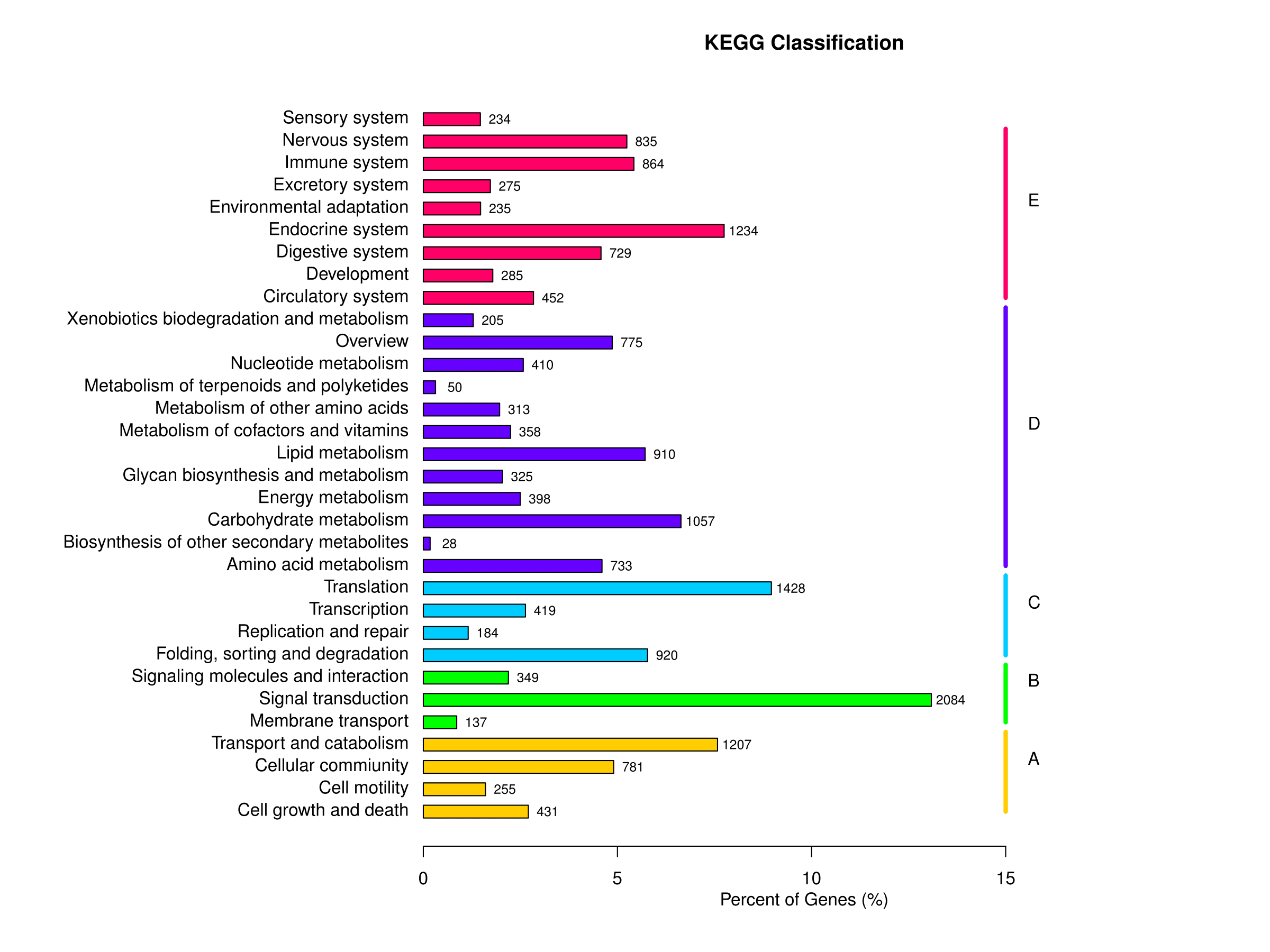


**Figure S8**. Kyoto Encyclopedia of Genes and Genomes (KEGG) annotation of the *A. swirskii* transcriptome. The top 15 pathways are shown. Y-axis is the names of KEGG pathways; X-axis is the number of the genes annotated in the pathway and the ratio between the number in this pathway and the total number of annotated genes. The KEGG metabolic pathways gene involved in are divided into 5 branches: A: Cellular Processes, B: Environmental Information Processing, C: Genetic Information Processing, D: Metabolism, E: Organismal Systems.

**Tables**

**Table S1**. Summary of the sequence reads obtained from eight transcriptome libraries of A. swirskii. Four transcriptome libraries are from female adults reared on tomato leaves (Treat_T1-Treat_T4) and four from females reared on pepper leaves (Treat_P1-Treat_P4).

| **Sample** | **Raw Reads** | **Clean Reads** | **Clean Bases** | **Error(%)** | **Q20(%)** | **Q30(%)** | **GC (%)** |
| --- | --- | --- | --- | --- | --- | --- | --- |
| Treat_T1 | 104344962 | 98272578 | 14.74G | 0.02 | 96.61 | 91.64 | 47.66 |
| Treat_T2 | 113731580 | 106805200 | 16.02G | 0.02 | 96.66 | 91.74 | 47.96 |
| Treat_T3 | 125843276 | 119195208 | 17.88G | 0.02 | 96.68 | 91.84 | 48.53 |
| Treat_T4 | 117805522 | 111478562 | 16.72G | 0.02 | 97.22 | 92.88 | 50.2 |
| Treat_P1 | 124524702 | 118665004 | 17.8G | 0.02 | 96.73 | 91.95 | 48.23 |
| Treat_P2 | 117971816 | 112701946 | 16.91G | 0.02 | 96.78 | 92.03 | 48.3 |
| Treat_P3 | 128821170 | 123369064 | 18.51G | 0.02 | 96.56 | 91.71 | 46.86 |
| Treat_P4 | 107798420 | 102843378 | 15.43G | 0.02 | 96.72 | 92.04 | 48.27 |
| Total | 940841448 | 893330940 | 134.01 | 0.02 | 96.75 | 91.98 | 48.25 |

**Table S2.** Summary of the assembled transcripts and unigenes obtained from the combined reads of eight transcriptome libraries of A. swirskii.

|  | **Transcripts** | **Unigenes** |  |
| --- | --- | --- | --- |
| Total number | 71345 | 71336 |  |
| Min length | 201 | 201 |  |
| Mean length | 1441 | 1441 |  |
| Median length | 914 | 914 |  |
| Max length | 21101 | 21101 |  |
| Total length | 102802715 | 102797693 |  |
| N50 | 2239 | 2239 |  |
| N90 | 620 | 620 |  |

**Table S3**. Summary of integrated annotations of unigenes

|  | **Number of Unigenes** | **Percentage (%)** |
| --- | --- | --- |
| Annotated in NR | 39574 | 55.47 |
| Annotated in NT | 18233 | 25.55 |
| Annotated in KO | 15927 | 22.32 |
| Annotated in SwissProt | 29384 | 41.19 |
| Annotated in PFAM | 34165 | 47.89 |
| Annotated in GO | 34270 | 48.04 |
| Annotated in KOG | 23070 | 32.33 |
| Annotated in all Databases | 7218 | 10.11 |
| Annotated in at least one Database | 47159 | 66.1 |
| Annotated in at least five Databases | 71336 | 100 |

**Table S4**. Classification of the CYP proteins of *A. swirskii.* used in the phylogenetic analysis. Columns are: Protein – assigned protein name; Accession No – the Accession number in SRA; Class – classification based on the results of the phylogenetic analysis shown in Figure X; NR Description, Hit Name, Organism, % Identity; and E-value – the results from the blastp against the NR database.

| **Protein** | **Accession No Cluster** | **Class** | **NR Description** | **Hit Name** | **Organism** | **% Identity** | **E Value** |
| --- | --- | --- | --- | --- | --- | --- | --- |
| AS_CYP_01 | 12473 | 4 | cytochrome P450 4c3 | XP_003744153 | *Galendromus occidentalis* | 72.4 | 0 |
| AS_CYP_02 | 14096.329 | 4 | cytochrome P450 4c3 | XP_003744153 | *Galendromus occidentalis* | 72.4 | 0 |
| AS_CYP_03 | 14096.47136 | 4 | cytochrome P450 4c3 | XP_003744153 | *Galendromus occidentalis* | 74.2 | 0 |
| AS_CYP_04 | 14096.47566 | 4 | cytochrome P450 4c3 | XP_003744153 | *Galendromus occidentalis* | 73.9 | 0 |
| AS_CYP_05 | 14096.14832 | 4 | cytochrome P450 4c3 | XP_003744153 | *Galendromus occidentalis* | 76.9 | 0 |
| AS_CYP_06 | 14096.38673 | 4 | uncharacterized protein | XP_018497071 | *Galendromus occidentalis* | 85.9 | 0 |
| AS_CYP_07 | 14096.39632 | 4 | uncharacterized protein | XP_018497071 | *Galendromus occidentalis* | 83.9 | 1.50E-95 |
| AS_CYP_08 | 14096.42635 | 4 | cytochrome P450 4c3 | XP_003741915 | *Galendromus occidentalis* | 79.0 | 9.30E-142 |
| AS_CYP_09 | 14096.46827 | 4 | cytochrome P450 4c3 | XP_003741915 | *Galendromus occidentalis* | 77.8 | 0 |
| AS_CYP_10 | 14096.29671 | 4 | cytochrome P450 4C1 | XP_003740735 | *Galendromus occidentalis* | 94.1 | 0 |
| AS_CYP_11 | 14096.32881 | 4 | cytochrome P450 4C1 | XP_003740735 | *Galendromus occidentalis* | 92.2 | 2.39E-68 |
| AS_CYP_12 | 14096.23635 | 4 | cytochrome P450 4V2-like | XP_003746392 | *Galendromus occidentalis* | 79.0 | 0 |
| AS_CYP_13 | 14096.32675 | 4 | cytochrome P450 4V2-like | XP_022657330 | Varroa destructor | 56.6 | 8.54E-131 |
| AS_CYP_14 | 14096.24998 | 4 | cytochrome P450 4V2-like | OQR78459 | Tropilaelaps mercedesae | 45.7 | 1.37E-164 |
| AS_CYP_15 | 14096.16794 | 4 | cytochrome P450 4V2-like | OQR73969 | Tropilaelaps mercedesae | 70.1 | 0 |
| AS_CYP_16 | 14096.17167 | 4 | cytochrome P450 4V2-like | XP_003744207 | *Galendromus occidentalis* | 67.2 | 0 |
| AS_CYP_17 | 14096.14981 | 4 | cytochrome P450 4V2 | XP_018493906 | *Galendromus occidentalis* | 88.2 | 0 |
| AS_CYP_18 | 14096.23655 | 4 | cytochrome P450 4V2 | XP_018493906 | *Galendromus occidentalis* | 67.3 | 0 |
| AS_CYP_19 | 14096.35067 | 4 | cytochrome P450 4V2-like | XP_018495014 | *Galendromus occidentalis* | 74.9 | 0 |
| AS_CYP_20 | 14096.39974 | 4 | uncharacterized protein | XP_003747976 | *Galendromus occidentalis* | 72.3 | 0 |
| AS_CYP_21 | 14096.28681 | 3 | cytochrome P450 3A28 | XP_003740908 | *Galendromus occidentalis* | 74.8 | 8.76E-137 |
| AS_CYP_22 | 14096.14914 | 3 | cytochrome P450 3A31 | XP_003744549 | *Galendromus occidentalis* | 80.9 | 0 |
| AS_CYP_23 | 14096.11337 | 3 | cytochrome P450 3A31 | XP_003744549 | *Galendromus occidentalis* | 62.2 | 7.04E-154 |
| AS_CYP_24 | 14096.22859 | 3 | cytochrome P450 3A6 | XP_018497017 | *Galendromus occidentalis* | 68.6 | 0 |
| AS_CYP_25 | 14096.12758 | 3 | cytochrome P450 3A6-like | XP_003745699 | *Galendromus occidentalis* | 65.1 | 0 |
| AS_CYP_26 | 14096.7201 | 3 | cytochrome P450 3A6-like | XP_003745699 | *Galendromus occidentalis* | 55.1 | 6.81E-130 |
| AS_CYP_27 | 14096.45017 | 3 | cytochrome P450 3A56 | XP_003744550 | *Galendromus occidentalis* | 54.8 | 0 |
| AS_CYP_28 | 14096.38234 | 3 | cytochrome P450 3A4 | XP_003743686 | *Galendromus occidentalis* | 78.0 | 0 |
| AS_CYP_29 | 14096.183 | 3 | cytochrome P450 3A24 | XP_003741765 | *Galendromus occidentalis* | 82.3 | 0 |
| AS_CYP_30 | 14096.23029 | 3 | cytochrome P450 3A24 | XP_003741765 | *Galendromus occidentalis* | 85.7 | 4.13E-137 |
| AS_CYP_31 | 14096.14727 | 3 | cytochrome P450 3A24 | XP_003741765 | *Galendromus occidentalis* | 83.8 | 3.41E-138 |
| AS_CYP_32 | 14096.37721 | 3 | cytochrome P450 3A31-like | XP_003744236 | *Galendromus occidentalis* | 72.5 | 0 |
| AS_CYP_33 | 14096.1855 | 3 | cytochrome P450 3A21 | XP_003741251 | *Galendromus occidentalis* | 81.9 | 2.52E-127 |
| AS_CYP_34 | 14096.2221 | 3 | cytochrome P450 3A21 | XP_003741251 | *Galendromus occidentalis* | 83.5 | 1.85E-112 |
| AS_CYP_35 | 14096.1389 | 3 | cytochrome P450 3A16-like | OQR73197 | Tropilaelaps mercedesae | 46.7 | 5.16E-71 |
| AS_CYP_36 | 14096.38954 | 3 | cytochrome P450 3A16-like | OQR73197 | Tropilaelaps mercedesae | 50.2 | 0 |
| AS_CYP_37 | 14096.18325 | 3 | cytochrome P450 3A16 | XP_003744817 | *Galendromus occidentalis* | 51.3 | 1.42E-30 |
| AS_CYP_38 | 14096.37108 | 3 | cytochrome P450 3A16 | XP_003744817 | *Galendromus occidentalis* | 51.2 | 2.13E-93 |
| AS_CYP_39 | 14096.8059 | 3 | cytochrome P450 3A16 | XP_003744817 | *Galendromus occidentalis* | 62.7 | 0 |
| AS_CYP_40 | 14096.17663 | 3 | cytochrome P450 3A9 | XP_003739531 | *Galendromus occidentalis* | 67.9 | 0 |
| AS_CYP_41 | 14096.39315 | 3 | cytochrome P450 3A24-like | XP_003746429 | *Galendromus occidentalis* | 70.6 | 0 |
| AS_CYP_42 | 14096.34995 | 3 | cytochrome P450 3A24-like | XP_003746429 | *Galendromus occidentalis* | 65.3 | 0 |
| AS_CYP_43 | 14096.16575 | 3 | cytochrome P450 3A24-like | XP_003746429 | *Galendromus occidentalis* | 63.2 | 3.89E-165 |
| AS_CYP_44 | 14096.40867 | 3 | probable cytochrome P450 6d5 | XP_003743386 | *Galendromus occidentalis* | 78.9 | 0 |
| AS_CYP_45 | 14096.18025 | 3 | cytochrome P450 3A29-like | XP_003737485 | *Galendromus occidentalis* | 72.5 | 0 |
| AS_CYP_46 | 14096.23742 | 3 | cytochrome P450 3A29-like | XP_003737485 | *Galendromus occidentalis* | 74.4 | 0 |
| AS_CYP_47 | 14096.22491 | 3 | cytochrome P450 3A29-like | XP_003737485 | *Galendromus occidentalis* | 64.8 | 0 |
| AS_CYP_48 | 14096.41971 | 3 | cytochrome P450 3A29-like | XP_003737485 | *Galendromus occidentalis* | 69.0 | 0 |
| AS_CYP_49 | 14096.19522 | 3 | cytochrome P450 3A9-like | XP_003738548 | *Galendromus occidentalis* | 59.4 | 0 |
| AS_CYP_50 | 14096.23153 | 3 | cytochrome P450 3A9-like | XP_003738548 | *Galendromus occidentalis* | 58.4 | 3.46E-167 |
| AS_CYP_51 | 14096.16757 | 3 | probable cytochrome P450 6a13 | XP_014094156 | Bactrocera oleae | 36.7 | 6.86E-17 |
| AS_CYP_52 | 14096.111 | M | cytochrome P450 302a1, mitochondrial-like | XP_003745070 | *Galendromus occidentalis* | 75.5 | 0 |
| AS_CYP_53 | 14096.16995 | M | probable cytochrome P450 49a1 | XP_003741296 | *Galendromus occidentalis* | 92.1 | 0 |
| AS_CYP_54 | 14096.41654 | 2 | cytochrome P450 2J6-like | XP_018494714 | *Galendromus occidentalis* | 66.9 | 0 |
| AS_CYP_55 | 14096.36233 | 2 | cytochrome P450 2J6-like | XP_018494714 | *Galendromus occidentalis* | 66.1 | 0 |
| AS_CYP_56 | 14096.22426 | 2 | cytochrome P450 2J6-like | XP_018494714 | *Galendromus occidentalis* | 64.6 | 0 |
| AS_CYP_57 | 14096.29831 | 2 | cytochrome P450 2C23 | XP_003747006 | *Galendromus occidentalis* | 76.3 | 0 |
| AS_CYP_58 | 14096.2349 | 2 | cytochrome P450 2J6 | XP_018495238 | *Galendromus occidentalis* | 91.4 | 0 |
| AS_CYP_59 | 14096.15944 | 2 | uncharacterized protein | XP_018494895 | *Galendromus occidentalis* | 57.7 | 0 |
| AS_CYP_60 | 14096.34069 | 2 | uncharacterized protein | XP_018494895 | *Galendromus occidentalis* | 59.7 | 0 |
| AS_CYP_61 | 14096.20597 | 2 | uncharacterized protein | XP_018494895 | *Galendromus occidentalis* | 69.7 | 2.20E-53 |
| AS_CYP_62 | 14096.33973 | 2 | uncharacterized protein | XP_018494895 | *Galendromus occidentalis* | 73.7 | 1.47E-179 |
| AS_CYP_63 | 14096.12347 | 2 | uncharacterized protein | XP_018494895 | *Galendromus occidentalis* | 63.2 | 1.34E-158 |
| AS_CYP_64 | 14096.19055 | 2 | cytochrome P450 1A1 | XP_003745229 | *Galendromus occidentalis* | 80.9 | 4.05E-165 |
| AS_CYP_65 | 14096.19304 | 2 | cytochrome P450 1A1 | XP_003745229 | *Galendromus occidentalis* | 81.7 | 0 |
| AS_CYP_66 | 14096.31074 | 2 | cytochrome P450 1A2 | XP_003741017 | *Galendromus occidentalis* | 64.8 | 5.33E-116 |
| AS_CYP_67 | 14096.24315 | 2 | cytochrome P450 2F3-like, partial | XP_003738046 | *Galendromus occidentalis* | 53.2 | 4.79E-162 |
| AS_CYP_68 | 14096.42315 | 2 | cytochrome P450 2J6-like | XP_003748194 | *Galendromus occidentalis* | 54.1 | 0 |
| AS_CYP_69 | 14096.16396 | 2 | cytochrome P450 2F3-like, partial | XP_003738046 | *Galendromus occidentalis* | 56.5 | 0 |
| AS_CYP_70 | 14096.44766 | 2 | cytochrome P450 2F3-like, partial | XP_003738046 | *Galendromus occidentalis* | 57.8 | 0 |
| AS_CYP_71 | 14096.16315 | 2 | cytochrome P450 2F3-like, partial | XP_003738046 | *Galendromus occidentalis* | 52.5 | 1.38E-180 |
| AS_CYP_72 | 14096.23357 | 2 | cytochrome P450 71B22-like | XP_003748195 | *Galendromus occidentalis* | 70.0 | 0 |
| AS_CYP_73 | 14096.20063 | 2 | cytochrome P450 2F3-like | XP_018493962 | *Galendromus occidentalis* | 54.9 | 0 |
| AS_CYP_74 | 14096.25695 | 2 | cytochrome P450 2F3-like | XP_018493962 | *Galendromus occidentalis* | 59.1 | 0 |
| AS_CYP_75 | 14096.16263 | 2 | cytochrome P450 307a1-like | XP_003747195 | *Galendromus occidentalis* | 83.0 | 0 |
| AS_CYP_76 | 14096.20839 | 2 | cytochrome P450 307a1-like | XP_003747195 | *Galendromus occidentalis* | 72.7 | 0 |

**Table S5**. Classification of the GST proteins of *A. swirskii.* used in the phylogenetic analysis. Columns are: Protein – assigned protein name; Accession No – the Accession number in SRA; Class – classification based on the results of the phylogenetic analysis shown in Figure X; NR Description, Hit Name, Organism, % Identity; and E-value – the results from the blastp against the NR database.

| **Protein** | **Accession No Cluster** | **Class** | **NR Description** | **Hit Name** | **Organism** | **% Identity** | **E Value** |
| --- | --- | --- | --- | --- | --- | --- | --- |
| AS_GST01 | 14096.28962 | omega | pyrimidodiazepine synthase | XP_018496135 | *Galendromus occidentalis* | 82.3 | 6.95E-140 |
| AS_GST02 | 14096.4445 | omega | glutathione S-transferase omega-1 | XP_003738156 | *Galendromus occidentalis* | 79.3 | 1.08E-90 |
| AS_GST03 | 14096.27508 | kappa | glutathione S-transferase kappa 1 | XP_003738807 | *Galendromus occidentalis* | 83.6 | 1.50E-139 |
| AS_GST04 | 14096.16668 | mu | glutathione S-transferase Mu 1-like | OQR71874 | Tropilaelaps mercedesae | 62.7 | 2.64E-50 |
| AS_GST05 | 14096.2226 | delta | glutathione S-transferase C-terminal domain-containing | XP_003737347 | *Galendromus occidentalis* | 69.7 | 0 |
| AS_GST06 | 14096.35417 | kappa | glutathione S-transferase kappa 1 | XP_003738778 | *Galendromus occidentalis* | 88.8 | 2.16E-151 |
| AS_GST07 | 14096.51772 | uncharacterized | glutathione S-transferase 4-like | XP_022669924 | Varroa destructor | 90.5 | 3.29E-125 |
| AS_GST08 | 14096.2419 | uncharacterized | glutathione S-transferase 4-like | XP_022669924 | Varroa destructor | 81.8 | 7.80E-121 |
| AS_GST09 | 14096.27038 | mu | glutathione S-transferase class-mu 26 kDa isozyme 47 | XP_003737612 | *Galendromus occidentalis* | 79.1 | 5.60E-60 |
| AS_GSTd01 | 14096.4354 | delta | glutathione S-transferase | EFX81633 | Daphnia pulex | 58.6 | 9.09E-69 |
| AS_GSTd02 | 14096.51347 | delta | glutathione S-transferase 1-1 | XP_003740940 | *Galendromus occidentalis* | 89.5 | 1.12E-137 |
| AS_GSTd03 | 14096.1759 | delta | glutathione S-transferase 1 | XP_003743487 | *Galendromus occidentalis* | 64.6 | 2.29E-55 |
| AS_GSTd04 | 14096.18035 | delta | glutathione S-transferase 1 | XP_003743487 | *Galendromus occidentalis* | 68.3 | 2.27E-103 |
| AS_GSTd05 | 14096.36333 | delta | glutathione S-transferase 1 | XP_003743487 | *Galendromus occidentalis* | 84.1 | 2.15E-102 |
| AS_GSTd06 | 14096.17204 | delta | glutathione S-transferase 1 | XP_003743487 | *Galendromus occidentalis* | 87.0 | 5.59E-136 |
| AS_GSTd07 | 14096.25844 | delta | glutathione S-transferase 1 | XP_003746787 | *Galendromus occidentalis* | 62.9 | 3.76E-78 |
| AS_GSTd08 | 14096.45023 | delta | glutathione S-transferase 1 | XP_003746787 | *Galendromus occidentalis* | 65.7 | 2.21E-105 |
| AS_GSTd09 | 14096.25851 | delta | glutathione S-transferase 1 | XP_003746787 | *Galendromus occidentalis* | 84.2 | 5.11E-138 |
| AS_GSTd10 | 14096.32345 | delta | glutathione S-transferase 1-like isoform X1 | XP_022705046 | Varroa jacobsoni | 68.4 | 9.71E-108 |
| AS_GSTd11 | 14096.33358 | delta | glutathione S-transferase 1 | XP_003746787 | *Galendromus occidentalis* | 78.7 | 4.24E-107 |
| AS_GSTm01 | 14096.8488 | mu | hypothetical protein PENSTE | OQE20378 | Penicillium steckii | 90.5 | 9.64E-133 |
| AS_GSTm02 | 14096.19298 | mu | glutathione S-transferase Mu 1 | XP_003742682 | *Galendromus occidentalis* | 68.8 | 1.08E-111 |
| AS_GSTm03 | 14096.27548 | mu | glutathione S-transferase Mu 1 | XP_003742682 | *Galendromus occidentalis* | 89.2 | 3.63E-146 |
| AS_GSTm04 | 14096.25965 | mu | glutathione S-transferase Mu 1 | XP_018495947 | *Galendromus occidentalis* | 89.1 | 4.05E-159 |
| AS_GSTm05 | 14096.24826 | mu | glutathione S-transferases-1 | AKO22175 | Dermanyssus gallinae | 57.4 | 1.07E-96 |
| AS_GSTo01 | 14096.47912 | omega | glutathione S-transferase omega-1 | XP_003738156 | *Galendromus occidentalis* | 82.5 | 2.77E-146 |
| AS_GSTo02 | 14096.46561 | omega | glutathione S-transferase omega-1 | XP_003738156 | *Galendromus occidentalis* | 80.8 | 9.58E-56 |
| AS_GSTo03 | 14096.2114 | omega | glutathione S-transferase omega-1 | XP_003746278 | *Galendromus occidentalis* | 77.7 | 1.64E-128 |

**Table S6**. Classification of the CCE proteins of *A. swirskii.* used in the phylogenetic analysis. Columns are: Protein – assigned protein name; Accession No – the Accession number in SRA; Class – classification based on the results of the phylogenetic analysis shown in Figure X; NR Description, Hit Name, Organism, % Identity; and E-value – the results from the blastp against the NR database.

| **Protein** | **Accession No Cluster** | **class** | **NR Description** | **Hit Name** | **Organism** | **% Identity** | **E Value** |
| --- | --- | --- | --- | --- | --- | --- | --- |
| AS_CCE_01 | 14096.17155 | J´´ | cholinesterase 1-like | XP_003744419 | *Galendromus occidentalis* | 72.9 | 0 |
| AS_CCE_02 | 14096.11228 | J´´ | cholinesterase 1-like | XP_003744359 | *Galendromus occidentalis* | 71.5 | 2.98E-154 |
| AS_CCE_03 | 14096.18193 | J´´ | liver carboxylesterase 1-like | OQR66681 | Tropilaelaps mercedesae | 53.1 | 4.09E-168 |
| AS_CCE_04 | 14096.38789 | J´´ | acetylcholinesterase-like | XP_003747077 | *Galendromus occidentalis* | 68.3 | 0 |
| AS_CCE_05 | 14096.7546 | J´´ | cholinesterase-like | XP_003740670 | *Galendromus occidentalis* | 42.0 | 8.67E-45 |
| AS_CCE_06 | 14096.854 | J´´ | acetylcholinesterase-like | XP_003745536 | *Galendromus occidentalis* | 52.3 | 1.31E-102 |
| AS_CCE_07 | 14096.26752 | J´´ | acetylcholinesterase-like | XP_003745536 | *Galendromus occidentalis* | 59.4 | 4.18E-141 |
| AS_CCE_08 | 14096.22721 | J´´ | cholinesterase 1-like | XP_003740671 | *Galendromus occidentalis* | 69.8 | 0 |
| AS_CCE_09 | 14096.48773 | J´´ | cholinesterase 1-like | XP_003740671 | *Galendromus occidentalis* | 56.7 | 5.96E-113 |
| AS_CCE_10 | 14096.6682 | J´´ | cholinesterase 1-like | XP_003740671 | *Galendromus occidentalis* | 62.1 | 0 |
| AS_CCE_11 | 14096.23959 | J´´ | cholinesterase 1-like | XP_003740671 | *Galendromus occidentalis* | 57.2 | 1.95E-130 |
| AS_CCE_12 | 14096.15138 | J´´ | cholinesterase 1-like | XP_003740671 | *Galendromus occidentalis* | 62.9 | 1.58E-99 |
| AS_CCE_13 | 14096.23398 | J´´ | cholinesterase 1-like | XP_003740671 | *Galendromus occidentalis* | 56.6 | 0 |
| AS_CCE_14 | 14096.32914 | J´´ | acetylcholinesterase-like | XP_003742289 | *Galendromus occidentalis* | 52.3 | 4.85E-102 |
| AS_CCE_15 | 14096.14165 | J´´ | carboxylesterase 5A-like | XP_003744189 | *Galendromus occidentalis* | 73.5 | 0 |
| AS_CCE_16 | 14096.6576 | J´´ | cholinesterase 2-like | XP_003744142 | *Galendromus occidentalis* | 74.1 | 0 |
| AS_CCE_17 | 14096.49601 | J´ | cholinesterase 1 | XP_003738701 | *Galendromus occidentalis* | 93.4 | 0 |
| AS_CCE_18 | 14096.44875 | J´ | acetylcholinesterase-1 | XP_003741414 | *Galendromus occidentalis* | 93.2 | 0 |
| AS_CCE_19 | 14096.42729 | J´ | acetylcholinesterase-1-like | XP_003743019 | *Galendromus occidentalis* | 89.7 | 0 |
| AS_CCE_20 | 14096.23198 | J´ | acetylcholinesterase-1-like | XP_003743019 | *Galendromus occidentalis* | 89.6 | 0 |
| AS_CCE_22 | 14096.17565 | J´ | acetylcholinesterase-like | XP_018497642 | *Galendromus occidentalis* | 91.3 | 0 |
| AS_CCE_23 | 14096.44036 | J´ | cholinesterase 1 | XP_003739903 | *Galendromus occidentalis* | 82.3 | 0 |
| AS_CCE_24 | 14096.6939 | J´ | cholinesterase 1 | XP_003739903 | *Galendromus occidentalis* | 84.8 | 0 |
| AS_CCE_25 | 14096.30297 | J´ | acetylcholinesterase-like | XP_018494386 | *Galendromus occidentalis* | 80.4 | 2.91E-78 |
| AS_CCE_26 | 14096.19664 | J´ | acetylcholinesterase-like | XP_018494387 | *Galendromus occidentalis* | 64.9 | 8.92E-112 |
| AS_CCE_27 | 14096.35506 | J´ | acetylcholinesterase-1-like | XP_018494388 | *Galendromus occidentalis* | 55.1 | 0 |
| AS_CCE_29 | 14096.11036 | J´ | acetylcholinesterase | XP_018496861 | *Galendromus occidentalis* | 87.5 | 0 |
| AS_CCE_30 | 14096.13369 | J´ | carboxylesterase 1C-like | XP_018494965 | *Galendromus occidentalis* | 92.0 | 0 |
| AS_CCE_31 | 14096.31091 | uncharacterized | cholinesterase-like | XP_003748221 | *Galendromus occidentalis* | 73.0 | 0 |
| AS_CCE_32 | 14096.22084 | uncharacterized | bile salt-activated lipase-like | XP_003748220 | *Galendromus occidentalis* | 54.8 | 1.92E-176 |
| AS_CCE_33 | 14096.17240 |  | neuroligin-4, Y-linked-like | XP_003747068 | *Galendromus occidentalis* | 97.1 | 6.48E-65 |
| AS_CCE_34 | 14096.919 |  | neuroligin-4, Y-linked-like | XP_003747068 | *Galendromus occidentalis* | 91.2 | 0 |
| AS_CCE_35 | 14096.48842 |  | neuroligin-4, Y-linked-like | XP_003747068 | *Galendromus occidentalis* | 91.4 | 0 |
| AS_CCE_36 | 14096.50683 |  | neuroligin-1-like | XP_003747809 | *Galendromus occidentalis* | 88.7 | 0 |
| AS_CCE_37 | 14096.34411 |  | neuroligin-1-like | XP_003746248 | *Galendromus occidentalis* | 96.6 | 6.62E-133 |
| AS_CCE_38 | 14096.10054 |  | neuroligin-2-like | XP_003740818 | *Galendromus occidentalis* | 50.7 | 7.05E-125 |
| AS_CCE_39 | 14096.612 |  | neuroligin-2-like | XP_003740818 | *Galendromus occidentalis* | 50.4 | 0 |
| AS_CCE_40 | 14096.617 |  | conserved hypothetical protein | XP_002433649 | *Ixodes scapularis* | 63.2 | 1.50E-117 |
| AS_CCE_41 | 14096.29182 |  | neuroligin-4, X-linked-like | XP_003740840 | *Galendromus occidentalis* | 86.5 | 0 |
| AS_CCE_42 | 14096.44003 |  | neuroligin-2-like | XP_003740818 | *Galendromus occidentalis* | 95.9 | 0 |
| AS_CCE_43 | 14096.43875 |  | neuroligin-4, X-linked-like | XP_003741692 | *Galendromus occidentalis* | 94.1 | 0 |
| AS_CCE_44 | 14096.7323 |  | neuroligin-2-like | XP_003742198 | *Galendromus occidentalis* | 83.4 | 0 |
| AS_CCE_45 | 14096.14167 |  | neuroligin-2-like | XP_003742198 | *Galendromus occidentalis* | 85.6 | 0 |
| AS_CCE_46 | 14096.46124 |  | neuroligin-2-like | XP_003742198 | *Galendromus occidentalis* | 89.9 | 0 |

**Table S7.** *G. occidentalis* CYP, GST and CCE gene accession numbers in GenBank, of the sequences used in the phylogenetic analyses.

| **Gene** | **Name in tree** | **Accession N.** |  |  | **Name in tree** | **Accession N.** |
| --- | --- | --- | --- | --- | --- | --- |
| **CYP** |  | **GenBank** |  |  |  | **GenBank** |
| 1 | MO_CYP1A1 | XP 003745229.1 |  | 31 | MO_CYP3A29-like | XP 003737485.1 |
| 2 | MO_CYP1A2 | XP 003741017.1 |  | 32 | MO_CYP3A30-like_partial | XP 018497550.1 |
| 3 | MO_CYP2B4-like | XP 018496574.1 |  | 33 | MO_CYP3A31 | XP 003744549.2 |
| 4 | MO_CYP2C23 | XP 003747006.1 |  | 34 | MO_CYP3A31-like | XP 003744236.1 |
| 5 | MO_CYP2C29 | XP 003747005.1 |  | 35 | MO_CYP3A40-like | XP 003743371.2 |
| 6 | MO_CYP2F2-like | XP 003738704.1 |  | 36 | MO_CYP3A56_ | XP 003744550.1 |
| 7 | MO_CYP2F3-like_ | XP 018493962.1 |  | 37 | MO_CYP4C1 | XP 003740735.2 |
| 8 | MO_CYP2J6 | XP 018495238.1 |  | 38 | MO_CYP4C1-like_1 | XP 018495350.1 |
| 9 | MO_CYP2J6-like_1 | XP 018494714.1 |  | 39 | MO_CYP4C1-like_2 | XP 003737352.2 |
| 10 | MO_CYP2J6-like_2 | XP 003748194.2 |  | 40 | MO_CYP4c3_1 | XP 003744153.1 |
| 11 | MO_CYP2J6-like_3 | XP 003748197.1 |  | 41 | MO_CYP4c3_2 | XP 003741915.1 |
| 12 | MO_CYP2U1-like | XP 003747196.2 |  | 42 | MO_CYP4c3-like_1 | XP 018497197.1 |
| 13 | MO_CYP3A4 | XP 003743686.1 |  | 43 | MO_CYP4c3-like_2 | XP 003739201.2 |
| 14 | MO_CYP3A6_1 | XP 018497017.1 |  | 44 | MO_CYP4V2_1 | XP 018493906.1 |
| 15 | MO_CYP3A6_2 | XP 003743392.2 |  | 45 | MO_CYP4V2_2 | XP 018496722.1 |
| 16 | MO_CYP3A6-like | XP 003745699.1 |  | 46 | MO_CYP4V2-like_1 | XP 003746392.1 |
| 17 | MO_CYP3A7-like | XP 018497741.1 |  | 47 | MO_CYP4V2-like_2 | XP 003744207.1 |
| 18 | MO_CYP3A9 | XP 003739531.2 |  | 48 | MO_CYP4V2-like_3 | XP 018495014.1 |
| 19 | MO_CYP3A9-like_ | XP 003738548.2 |  | 49 | MO_CYP4V2-like_4 | XP 003747974.1 |
| 20 | MO_CYP3A12-like | XP 003744583.1 |  | 50 | MO_CYP4V2-like_5 | XP 018494969.1 |
| 21 | MO_CYP3A14 | XP 003745878.1 |  | 51 | MO_CYP71B22-like | XP 003748195.1 |
| 22 | MO_CYP3A14-like | XP 003743440.1 |  | 52 | MO_CYP98A3-like_ | XP 018495925.1 |
| 23 | MO_CYP3A16 | XP 003744817.1 |  | 53 | MO_CYP302a1_mitochondrial-like | XP 003745070.1 |
| 24 | MO_CYP3A19-like_1 | XP 003742033.2 |  | 54 | MO_CYP307a1-like | XP 003747195.1 |
| 25 | MO_CYP3A19-like_2 | XP 018496532.1 |  | 55 | MO_CYP315a1_mitochondrial | XP 003743235.1 |
| 26 | MO_CYP3A21 | XP 003741251.1 |  | 56 | MO_CYP_6d5_ | XP 003743386.2 |
| 27 | MO_CYP3A24 | XP 003741765.1 |  | 57 | MO_CYP_49a1_ | XP 003741296.1 |
| 28 | MO_CYP3A24-like_1 | XP 003746429.1 |  | 58 | MO_ecdysone_20-monooxygenase-like | XP 003748577.1 |
| 29 | MO_CYP3A24-like_2 | XP 003738840.2 |  | 59 | MO_lithocholate_6-beta-hydroxylase-like | XP 003745726.1 |
| 30 | MO_CYP3A28 | XP 003740908.1 |  |  |  |  |
| **GST** |  |  |  |  |  |  |
| 1 | MO_GST05 | XP_003738807.1 |  | 10 | MO_GST04 | XP 003744624.1 |
| 2 | MO_GST06 | XP_003738778.1 |  | 11 | MO_GST03 | XP 003741590.1 |
| 3 | MO_GSTp01 | XP 003737007.1 |  | 12 | MO_GST01 | XP 003738383.1 |
| 4 | MO_GSTo01 | XP 003746278.1 |  | 13 | MO_GSTp02 | XP 003744621.1 |
| 5 | MO_GSTo02 | XP 003738156.1 |  | 14 | MO_GSTm01 | XP 003737612.1 |
| 6 | MO_GSTd01 | XP 003740940.1 |  | 15 | MO_GSTm02 | XP 003742682.2 |
| 7 | MO_GSTd02 | XP 003743487.1 |  | 16 | MO_GSTm03 | XP 018495947.1 |
| 8 | MO_GSTd03 | XP 003746787.1 |  | 17 | MO_GSTm04 | XP 003747409.1 |
| 9 | MO_GST02 | XP 003745766.1 |  | 18 | MO_GST07 | XP 003744890.1 |
| **CCE** |  |  |  |  |  |  |
| 1 | MO CCE3 | XP 003744359.1 |  | 24 | MO CCE37 | XP 003748221.1 |
| 2 | MO CCE4 | XP 003744419.1 |  | 25 | MO CCE38 | XP 003748220.1 |
| 3 | MO CCE5 | XP 003740650.1 |  | 26 | MO CCE39 | XP 018497642.1 |
| 4 | MO CCE6 | XP 003740671.2 |  | 27 | MO CCE40 | XP 018496563.1 |
| 5 | MO CCE7 | XP 003740670.1 |  | 28 | MO CCE41 | XP 003737110.1 |
| 6 | MO CCE8 | XP 003745536.1 |  | 29 | MO CCE42 | XP 003738697.1 |
| 7 | MO CCE9 | XP 003747077.1 |  | 30 | MO CCE43 | XP 028966555.1 |
| 8 | MO CCE10 | XP 003744142.2 |  | 31 | MO CCE44 | XP 018494965.1 |
| 9 | MO CCE12 | XP 003744189.1 |  | 32 | MO CCE45 | XP 018496352.1 |
| 10 | MO CCE13 | XP 003742834.1 |  | 33 | MO CCE46 | XP 018494388.1 |
| 11 | MO CCE14 | XP 003738421.1 |  | 34 | MO CCE47 | XP 003739903.2 |
| 12 | MO CCE18 | XP 003745369.1 |  | 35 | MO CCE48 | XP 028968333.1 |
| 13 | MO CCE20 | XP 003744479.1 |  | 36 | MO CCE49 | XP 018496861.1 |
| 14 | MO CCE22 | XP 003746194.2 |  | 37 | MO CCE50 | XP 018496632.1 |
| 15 | MO CCE26 | XP 003743019.1 |  | 38 | MO CCE51 | XP 018494964.1 |
| 16 | MO CCE27 | XP 003738701.1 |  | 39 | MO CCE52 | XP 018494461.1 |
| 17 | MO CCE28 | XP 003741414.1 |  | 40 | MO CCE53 | XP 028966956.1 |
| 18 | MO CCE30 | XP 003747841.1 |  | 41 | MO CCE54 | XP 028966822.1 |
| 19 | MO CCE31 | XP 003739668.2 |  | 42 | MO CCE55 | XP 028966555.1 |
| 20 | MO CCE32 | XP 003742457.2 |  | 43 | MO CCE56 | XP 028966562.1 |
| 21 | MO CCE33 | XP 003745863.1 |  | 44 | MO CCE57 | XP 028968576.1 |
| 22 | MO CCE34 | XP 003743644.1 |  | 45 | MO CCE58 | XP 028968124.1 |
| 23 | MO CCE35 | XP 003746193.1 |  | 46 | MO CCE59 | XP 028968529.1 |

**Table S8.** *T. urticae* CYP, GST and CCE gene accession numbers in OrcAE, of the sequences used in the phylogenetic analyses.

| **Gene** | **Name in tree** | **Accession N.** |  |  | **Name in tree** | **Accession N.** |
| --- | --- | --- | --- | --- | --- | --- |
| **CYP** |  | **OrcAE** |  |  |  | **OrcAE** |
| 1 | TU_CYP4CF2 | tetur09g03800 |  | 44 | TU_CYP392A6 | tetur11g00530 |
| 2 | TU_CYP4CL1 | tetur01g00650 |  | 45 | TU_CYP392A7 | tetur16g03500 |
| 3 | TU_CYP302A1_1 | tetur05g02550 |  | 46 | TU_CYP392A8 | tetur02g14020 |
| 4 | TU_CYP302A1_2 | tetur05g02670 |  | 47 | TU_CYP392A9 | tetur47g00090 |
| 5 | TU_CYP307A1 | tetur10g03900 |  | 48 | TU_CYP392A9v2 | tetur02g14330 |
| 6 | TU_CYP314A1 | tetur03g03020 |  | 49 | TU_CYP392A10 | tetur16g03790 |
| 7 | TU_CYP315A1 | tetur06g05620 |  | 50 | TU_CYP392A10v2 | tetur02g14400 |
| 8 | TU_CYP381A1 | tetur13g02850 |  | 51 | TU_CYP392A11 | tetur03g00970 |
| 9 | TU_CYP381A2 | tetur13g02840 |  | 52 | TU_CYP392A12 | tetur03g00830 |
| 10 | TU_CYP382A1 | tetur03g01560 |  | 53 | TU_CYP392A13v1 | tetur08g08050 |
| 11 | TU_CYP384A1 | tetur38g00650 |  | 54 | TU_CYP392A13v2 | tetur03g00020 |
| 12 | TU_CYP385A1 | tetur07g05500 |  | 55 | TU_CYP392A14 | tetur08g07950 |
| 13 | TU_CYP385B1 | tetur05g04000 |  | 56 | TU_CYP392A16 | tetur06g04520 |
| 14 | TU_CYP385C1 | tetur26g01470 |  | 57 | TU_CYP392B1 | tetur20g03200 |
| 15 | TU_CYP385C2 | tetur11g05000 |  | 58 | TU_CYP392B2 | tetur02g06650 |
| 16 | TU_CYP385C3 | tetur11g05540 |  | 59 | TU_CYP392B3 | tetur20g00290 |
| 17 | TU_CYP385C3v2 | tetur46g00170 |  | 60 | TU_CYP392C1 | tetur03g03950 |
| 18 | TU_CYP385C4 | tetur11g05520 |  | 61 | TU_CYP392D1 | tetur23g00260 |
| 19 | TU_CYP385C4v2 | tetur46g00150 |  | 62 | TU_CYP392D2 | tetur03g04990 |
| 20 | TU_CYP386A1 | tetur11g06070 |  | 63 | TU_CYP392D3 | tetur03g05000 |
| 21 | TU_CYP387A1 | tetur08g06170 |  | 64 | TU_CYP392D4 | tetur03g05010 |
| 22 | TU_CYP387A2 | tetur01g06120 |  | 65 | TU_CYP392D6 | tetur03g05030 |
| 23 | TU_CYP388A1 | tetur03g05190 |  | 66 | TU_CYP392D8 | tetur03g05070 |
| 24 | TU_CYP389A1 | tetur25g02050 |  | 67 | TU_CYP392E1 | tetur03g05540 |
| 25 | TU_CYP389B1 | tetur25g02060 |  | 68 | TU_CYP392E2 | tetur06g02400 |
| 26 | TU_CYP389C1 | tetur34g00510 |  | 69 | TU_CYP392E3 | tetur06g02820 |
| 27 | TU_CYP389C2 | tetur05g02950 |  | 70 | TU_CYP392E6 | tetur27g00330 |
| 28 | TU_CYP389C3 | tetur05g02960 |  | 71 | TU_CYP392E7 | tetur27g00340 |
| 29 | TU_CYP389C4 | tetur05g02970 |  | 72 | TU_CYP392E8 | tetur27g00350 |
| 30 | TU_CYP389C5 | tetur05g08390 |  | 73 | TU_CYP392E9 | tetur27g01020 |
| 31 | TU_CYP389C6 | tetur05g06630 |  | 74 | TU_CYP392E10 | tetur27g01030 |
| 32 | TU_CYP389C7 | tetur05g06620 |  | 75 | TU_CYP406A1 | tetur01g04440 |
| 33 | TU_CYP389C8 | tetur05g06610 |  | 76 | TU_CYP407A1 | tetur20g00830 |
| 34 | TU_CYP389C9 | tetur05g06600 |  | 77 | TU_CYP_1 | tetur14g01350 |
| 35 | TU_CYP389C10 | tetur05g06580 |  | 78 | TU_CYP_2 | tetur05g02670 |
| 36 | TU_CYP389C11 | tetur05g06570 |  | 79 | TU_CYP_3 | tetur01g13730 |
| 37 | TU_CYP389C12 | tetur05g06560 |  | 80 | TU_CYP_conserved_site_1 | tetur03g09941 |
| 38 | TU_CYP390A1 | tetur03g00910 |  | 81 | TU_CYP_conserved_site_2 | tetur03g09961 |
| 39 | TU_CYP391A1 | tetur36g00920 |  | 82 | TU_CYP_conserved_site_3 | tetur03g05100 |
| 40 | TU_CYP392A1 | tetur07g06410 |  | 83 | TU_CYP_E-class_group_I | tetur06g02650 |
| 41 | TU_CYP392A3 | tetur07g06460 |  | 84 | TU_CYP_E-class_group_I_1 | tetur03g00970 |
| 42 | TU_CYP392A4 | tetur07g06480 |  | 85 | TU_CYP_E-class_group_I_2 | tetur27g02598 |
| 43 | TU_CYP392A5 | tetur11g04390 |  | 86 | TU_CYP_E-class_group_IV | tetur07g08087 |
| **GST** |  |  |  |  |  |  |
| 1 | TU GSTp01 | tetur05g05180 |  | 18 | TU GSTd13 | tetur26g01490 |
| 2 | TU GSTd15 | tetur26g02802 |  | 19 | TU GSTd05 | tetur01g02470 |
| 3 | TU GSTd12 | tetur26g02801 |  | 20 | TU GSTo02 | tetur01g02320 |
| 4 | TU GSTm05 | tetur05g05270 |  | 21 | TU GSTd03 | tetur29g00220 |
| 5 | TU GSTm07 | tetur05g05210 |  | 22 | TU GSTz01 | tetur07g02560 |
| 6 | TU GSTd08 | tetur03g07920 |  | 23 | TU GSTd07 | tetur01g02500 |
| 7 | TU GSTd10 | tetur26g01450 |  | 24 | TU GSTm10 | tetur05g05240 |
| 8 | TU GSTm03 | tetur03g09230 |  | 25 | TU GSTd05 | tetur01g02480 |
| 9 | TU GSTd01 | tetur31g01390 |  | 26 | TU GSTm11 | tetur05g05190 |
| 10 | TU GST02 | tetur01g12390 |  | 27 | TU GSTd14 | tetur26g01510 |
| 11 | TU GST01 | tetur04g04990 |  | 28 | TU GSTk01 | tetur22g02300 |
| 12 | TU GSTm09 | tetur05g05260 |  | 29 | TU GSTm01 | tetur05g05220 |
| 13 | TU GSTd02 | tetur31g01330 |  | 30 | TU GSTm04 | tetur05g05200 |
| 14 | TU GSTd06 | tetur01g02230 |  | 31 | TU GSTo01 | tetur12g03900 |
| 15 | TU GSTm08 | tetur05g05250 |  | 32 | TU GSTm02 | tetur05g05300 |
| 16 | TU GSTm06 | tetur05g05290 |  | 33 | TU GSTd04 | tetur01g02510 |
| 17 | TU GSTd11 | tetur26g01500 |  | 34 | TU GSTd09 | tetur26g01460 |
| **CCE** |  |  |  |  |  |  |
| 1 | TU_CCE_1 | tetur01g10760 |  | 6 | TU_CCE_6 | tetur04g08480 |
| 2 | TU_CCE_2 | tetur01g10830 |  | 7 | TU_CCE_7 | tetur11g05770 |
| 3 | TU_CCE_3 | tetur01g14090 |  | 8 | TU_CCE_8 | tetur19g00850 |
| 4 | TU_CCE_4 | tetur02g06930 |  | 9 | TU_CCE_9 | tetur29g00970 |
| 5 | TU_CCE_5 | tetur04g06380 |  | 10 | TU_CCE_10 | tetur30g01560 |

**Table S9.** *A. meliphera* (left columns) and *D. melanogaster* (right columns) CCE gene accession numbers in GenBank and FlyBase respectively, of the sequences used in the phylogenetic analyses.

| **Gene** | **Name in tree** | **Accession N.** |  |  | **Name in tree** | **Accession N.** |
| --- | --- | --- | --- | --- | --- | --- |
| **CCE** |  | **GenBank** |  |  |  | **FlyBase** |
| 1 | AM CCE 1 | NP 001119716.1 |  | 1 | DM CCE 1 | CG2505 |
| 2 | AM AChE1 | XP_393751.2 |  | 2 | DM CCE 2 | CG17148 |
| 3 | AM CCE 3 | XP 006572393.1 |  | 3 | DM CCE 3 | CG31146 |
| 4 | AM CCE 4 | XP 006568129.1 |  | 4 | DM CCE 4 | CG1121 |
| 5 | AM CCE 5 | XP 006564306.1 |  | 5 | DM CCE 5 | CG9704 |
| 6 | AM CCE 6 | NP 001035320.1 |  | 6 | DM CCE 6 | CG9280 |
| 7 | AM CCE 7 | XP 016768436.1 |  | 7 | DM CCE 7 | CG12869 |
| 8 | AM CCE 8 | XP 006566930.1 |  | 8 | DM CCE 8 | CG6917 |
| 9 | AM CCE 9 | XP 006571679.2 |  | 9 | DM CCE 9 | CG9289 |
|  |  |  |  | 10 | DM CCE 10 | CG9287 |
|  |  |  |  | 11 | DM CCE 11 | CG17907 |
|  |  |  |  | 12 | DM CCE 12 | CG3903 |
|  |  |  |  | 13 | DM CCE 13 | CG8425 |
|  |  |  |  | 14 | DM CCE 14 | CG13772 |
|  |  |  |  | 15 | DM CCE 15 | CG5397 |
